# The Interplay between the Immune and the Endocannabinoid Systems in Cancer

**DOI:** 10.3390/cells10061282

**Published:** 2021-05-21

**Authors:** Mariantonia Braile, Simone Marcella, Gianni Marone, Maria Rosaria Galdiero, Gilda Varricchi, Stefania Loffredo

**Affiliations:** 1 Department of Translational Medical Sciences, Center for Basic and Clinical Immunology Research (CISI), University of Naples Federico II, 80131 Naples, Italy; brailemariantonia@gmail.com (M.B.); s.marcella92@gmail.com (S.M.); marone@unina.it (G.M.); mrgaldiero@libero.it (M.R.G.); 2 WAO Center of Excellence, 80131 Naples, Italy; 3 Institute of Experimental Endocrinology and Oncology (IEOS), National Research Council (CNR), 80131 Naples, Italy

**Keywords:** angiogenesis, cancer, cannabis, endocannabinoid system, immune cells, tumor microenvironment

## Abstract

The therapeutic potential of *Cannabis sativa* has been recognized since ancient times. Phytocannabinoids, endocannabinoids and synthetic cannabinoids activate two major G protein-coupled receptors, subtype 1 and 2 (CB1 and CB2). Cannabinoids (CBs) modulate several aspects of cancer cells, such as apoptosis, autophagy, proliferation, migration, epithelial-to-mesenchymal transition and stemness. Moreover, agonists of CB1 and CB2 receptors inhibit angiogenesis and lymphangiogenesis in vitro and in vivo. Low-grade inflammation is a hallmark of cancer in the tumor microenvironment (TME), which contains a plethora of innate and adaptive immune cells. These cells play a central role in tumor initiation and growth and the formation of metastasis. CB2 and, to a lesser extent, CB1 receptors are expressed on a variety of immune cells present in TME (e.g., T cells, macrophages, mast cells, neutrophils, NK cells, dendritic cells, monocytes, eosinophils). The activation of CB receptors modulates a variety of biological effects on cells of the adaptive and innate immune system. The expression of CB2 and CB1 on different subsets of immune cells in TME and hence in tumor development is incompletely characterized. The recent characterization of the human cannabinoid receptor CB2-G_i_ signaling complex will likely aid to design potent and specific CB2/CB1 ligands with therapeutic potential in cancer.

## 1. Introduction

The therapeutic properties of *Cannabis sativa* have been recognized since ancient times. *Cannabis* was used during the Han Chinese dynasty to treat inflammatory disorders and malaria [1,2]. Other ancient people used *Cannabis* for healing and recreation in several regions of the world [3,4]. In the western world, the importance of *Cannabis* was recognized in the mid-19th century, when an Irish physician, William B. O’Shaughnessy, and a French psychiatrist, Jacques-Joseph Moreau, reported that *Cannabis* had some beneficial effects [5,6]. *Cannabis* was also used for religious and textile purposes [3,5]. More recently, *Cannabis* was used as a recreational drug and the emergent problem of opiate dependency caused legal restrictions in 1925. In 1941, the American National Formulary and Pharmacopoeia included these compounds among illicit drugs [6,7]. In recent years, there have been legislative changes to allow the use of *Cannabis* for some medical and/or recreational purposes [8,9]. In this review we describe the interplay between endocannabinoids and immune systems in the cancer context.

## 2. The Endocannabinoid System (ECS)

*Cannabis* contains more than 100 molecules which are known as phytocannabinoids [4]. In the 1990s, two cannabinoid receptors (CB1 and CB2) [10,11] and their endogenous ligands, which are known as endocannabinoids (ECs) were identified [12,13,14,15]. Subsequently, the enzymes involved in endocannabinoid biosynthesis and inactivation were identified [16,17]. Collectively, this complex system is known as the endocannabinoid system (ECS) or the endocannabinoidome [18].

*Phytocannabinoids*. Phytocannabinoids, produced by *Cannabis* plants, are a group of terpene phenolic compounds composed of 21-carboxylated carbon or 22-carboxylated carbon [19]. More than 100 plant cannabinoids (CBs) have been isolated and characterized [19]. Δ^9^-tetrahydrocannabinol (Δ^9^-THC) was the main psychoactive CB isolated from *Cannabis* [20]. This compound induces euphoria, analgesia [21], appetite stimulation [22], and exerts antiemetic and anti-inflammatory effects [23]. Several non-psychotropic phytocannabinoids have currently been defined as constituents of *Cannabis* [24]. Among these compounds there are cannabinol and cannabidiol (CBD) [25]. CBD exerts anti-inflammatory, analgesic and antianxiety activities [26]. In contrast to Δ9-THC, which possesses both therapeutic effects and some important adverse effects, CBD is more tolerated and has several therapeutic properties, including antitumoral properties [26,27]. 

*Endocannabinoids (ECs).* ECs are lipid molecules containing long-chain polyunsaturated fatty acids, amides, esters and ethers. They exert their biological effects through the engagement of CB receptors and non-CB receptors [20]. ECs are neuromodulators [18] and modulate inflammation, fat and energy metabolism [28]. The first EC discovered in humans was an amide of arachidonic acid and ethanolamine, N-arachidonoylethanolamide (anandamide, AEA) [12]. 2-arachidonoylglycerol (2-AG) was discovered in greater quantities in the brain where the CB1 receptor is highly expressed [13,14]. AEA and 2-AG are not only produced by neurons but also by certain immune cells [29,30]. 

In mammals, the ECS includes CB1 and CB2 receptors, endogenous ligands and enzymes responsible for their metabolism and transport [31,32,33]. Although the ECS is highly expressed in the nervous and immune systems, it is present in almost all organs of the body [34]. The ECS is physiologically important, and its alteration is involved in neurodegenerative disorders, inflammation, cardiovascular disease, obesity and cancer [33,35]. Lipids similar to endogenous CB receptor ligands, such as oleoyl- and palmitoyl-ethanolamide (OEA and PEA) are included in the definition of ECS [36]. PEA, released by the immune cells downregulates the inflammatory process [37,38] through the activation of CB2 receptor. Other compounds such as 2-AG-ether and O-arachidonoylethanolamine [39,40] also belong to the EC family.

*Synthetic cannabinoids (SCBs).* SCBs are produced by chemical synthesis [41,42]. These compounds bind to the CB receptors and produce effects similar to those induced by phytocannabinoids and ECs [41,43]. Certain SCBs show greater selectivity and potency compared to natural ligands [41,43]. The increase in their use as a recreational drug [44] and the adverse health effects of SCBs (e.g., tachycardia, breathing disorders, and seizures) have led to concerns globally [44,45]. More than 100 compounds are SCBs, including ACEA (CB1 agonist) and JWH-133 (CB2 agonist) [29,46]. Based on their chemical structure they can be divided into four classes: aminoalkylindoles, classic CB, non-classic CB and fatty acid amides [47]. Depending on the type of agonist, target tissues, route of administration, dose and duration of treatment, SCBs can cause inhibition of cell growth and proliferation, inhibition of viability, and inhibition of the release of proinflammatory cytokines [44] and angiogenic factors [29,46]. Some of these compounds may represent a promising therapeutic approach for cancer [43,48].

### 2.1. Receptors 

*Classical receptors.* CB1 and CB2 receptors belong to the family of G protein-coupled receptor (GPCRs) [10,11]. The activation of these receptors inhibits adenylyl cyclase, resulting in reduced cytoplasmic cyclic adenosine monophosphate (cAMP) production, closure of the Ca^2+^ channel, and stimulation of protein kinases that play a key role in multiple signaling pathways, including mitogen-activated protein kinase (MAPK), phosphoinositide 3-kinase (PI3K), or cyclooxygenase (COX) 2 pathways [49].

Although the CB1 receptor is ubiquitous, the expression of this receptor is greatest within the central nervous system (CNS) [50]. CB1 receptors are also expressed by certain immune cells [29,46,51]. CB2 receptors are found mainly on several immune cells, although their expression has also been observed in the CNS [51,52]. Endocannabinoids bind to both CB receptors; AEA has a higher affinity for the CB1 receptor, while 2-AG has the same affinity for both CB receptors [53]. CB agonists are involved in several neurological disorders [18] and in various models of cancer [54,55,56,57].

#### Other Endocannabinoidome Receptors

The orphan G protein-coupled receptor 55 (GPR55) is considered a full-fledged CB receptor [58]. Although its endogenous ligand is the phospholipid lysophosphatidylinositol [59], AEA, 2-AG and PEA can activate this receptor [47,60]. In breast cancer, GPR55 has been found to heterodimerize with CB receptors and its targeting reduces tumor growth [49]. Furthermore, heterodimerization of CB2 receptor with HER2 and with C-X-C chemokine receptor type 4 (CXCR4) causes the activation of CB2 and inhibits HER2 and CXCR4 signaling. It has been suggested that this condition may represent a promising target in antitumor strategy [61,62].

In recent years, new groups of receptors, potentially belonging to the family of non-classical EC receptors, have been described [63,64,65]. Among these, there is a large superfamily of transient receptor potential (TRP). These are non-selective cation channels including the transient vanilloid type 1 (TRPV1), a capsaicin receptor and TRPV2 [66,67]. AEA and CBD activate TRPV1 channels [18,68]. Another potential part of the ECS is the group of nuclear receptors called peroxisome proliferator-activated nuclear receptor-α (PPARα) and PPARγ [69]. 2-AG, and AEA can activate PPARα and PPARγ, respectively [18]. Although the detailed mechanisms of cannabinoid-PPAR interactions are not completely elucidated, there is some evidence that PPAR are also involved in immune cell regulation. [70].

### 2.2. Enzymes

Arachidonic acid is the initial substrate for the biosynthesis of AEA and 2-AG [39]. These compounds can also be stored in intracellular vesicles or organelles [71]. Enzymes involved in the synthesis and degradation of ECs play an important role in cell signal transduction [72]. Both AEA and 2-AG are removed from their sites of action by uptake (e.g., simple diffusion, membrane-associated binding proteins or cellular transmembrane transporter protein) and metabolized intracellularly [73]. The enzymes involved in the degradation of ECs are fatty acid-amide hydrolase (FAAH) and monoacylglycerol lipase (MAGL) [23,74,75,76,77]. FAAH and MAGL represent possible therapeutic targets for the treatment of several disorders [70]. In some tissues, ECs can also undergo oxidative catabolism through the lipoxygenase, cyclooxygenase 2 (COX-2), and cytochrome P450 isoenzymes [78]. The action of these enzymes leads to the generation of various compounds such as prostaglandin-ethanolamides and glyceryl esters, hydroxy-anandamides and hydroxycosatetraenoylglycerols [79]. Some of these endocannabinoid metabolites are biologically active [80].

## 3. Cannabinoids and Tumorigenesis

There is growing interest in the roles of CBs in the modulation of various aspects of cancer growth [4,81]. Cancer cells rapidly and uncontrollably proliferate and have the ability to invade other tissues, causing metastasis [82]. It has been reported that ECS dysregulation occurs during carcinogenesis and may be responsible for cancer aggressiveness [83,84,85,86,87]. ECs can modulate several aspects of tumorigenesis [88,89,90,91]. A major discovery was the recognition of the ability of CBs to kill a plethora of cancer cells (e.g., lung, skin, breast, prostate and pancreatic cancer, glioma) [92]. CBs promote apoptosis and autophagy, induce cell cycle arrest, and have inhibitory effects on the migration, invasion, and self-renewal of tumor cells [68,88,90,91]. These processes can be both dependent and independent of CB receptors, showing that the antitumor activity of CBs has much more complex molecular mechanisms than originally thought [88]. In addition, CBs exhibit several palliative effects in cancer patients (e.g., inhibition of nausea and vomiting, stimulation of appetite, pain relief, mood elevation, and relief from insomnia) [4,93,94].

*Apoptosis, autophagy and inhibition of proliferation.* A major characteristic of cancer cells is uncontrolled proliferation. CB receptor activation increases de novo production of ceramide, a sphingolipid with proapoptotic functions [92,95]. The upregulation of the ceramide-induced stress-regulated protein p8 causes apoptosis through the overexpression of genes encoding the activating transcription factor 4, and Tribbles homolog 3 (TRB3) [49,96]. TRB3 is also responsible for inhibition of the Akt/mTORC1 complex axis, which causes autophagy-mediated apoptosis and the inhibition of cell proliferation [68,72,97].

In Jurkat leukemia T cells, Δ^9^-THC leads to apoptosis through the downregulation of Raf-1, a kinase of extracellular signal-regulated kinase 1 and 2 (ERK1/2) pathway [98]. In experimental colorectal cancer, apoptosis can be caused by the downregulation of another member of ERK1/2 pathway, Ras, and PI3K-Akt survival pathway [99]. CBs also promote apoptosis through inhibition of mitochondrial metabolism, increase of reactive oxygen species (ROS) production and caspase activation [100], and induce autophagy [101,102,103,104]. CBs also cause apoptosis and proliferative arrest by inhibiting the signaling of the pancreatic beta cell insulin receptor, which shows a direct interaction with CB1 receptor via Akt, MAPK and ERK pathways [105,106]. It has been reported that TRPV2 is a cannabinoid target involved in CBD-induced autophagy [107].

CBs cause an overexpression of p21_waf_ and p27_kip1_, blocking the cell cycle in the G1/S transition and the cyclin-dependent kinase (Cdk) 2 complex [49,68]. This overexpression of p21_waf_ leads to a G2/M cell cycle arrest [49,68]. Cell cycle arrest in G2/M phase is also caused by downregulation of the Cdk1 protein [108] or modulation of JunD [109]. Interestingly, in cancer cells this mechanism is upregulated in several signaling pathways [110]. In breast cancer the anti-proliferative effects are induced by inhibition of epidermal growth factor (EGF), NF-kB, ERK/Akt and matrix metalloproteinase (MMP) 2 and 9 signaling pathways [111].

*Migration and invasion.* Cancer cells migrate more than normal cells, and they spread to surrounding tissues and distant organs, leading to the formation of metastases [112]. There is ample evidence that the activation of CB receptors can modulate these processes [113,114,115,116,117,118,119]. Activation of CB receptors impairs cancer cell invasion through the release of tissue inhibitors of metalloproteinases (TIMP) 1 and TIMP-4 and down-regulation of MMP-2 [68,111,120].

In lung cancer, CB treatment inhibits the EGF-induced phosphorylation of ERK1/2, c-Jun-NH2-kinase1/2 and Akt, causing metastatic and proliferative suppression [61,121]. In addition, CB receptor activation inhibits the phosphorylation of ERK and CXCR4 polymerization, causing CXCL12 suppression and consequently cell migration and invasion [122].

Epithelial-to-mesenchymal transition (EMT) and stemness are fundamental features of cancer cells [123]. CBs block the Wnt/β-catenin pathway inducing a decrease of EMT and a reduction of mesenchymal markers (e.g., vimentin) [91,124] favoring a consequent inhibition of migration, invasion, angiogenesis and finally self-renewal of cancer stem cells (CSCs) [91,124,125,126,127]. CB receptors, also expressed in glioblastoma stem-like cells (GSCs), promote neural differentiation of GSC and inhibit gliomagenesis [128]. The effects of CB on stem cells, and consequently on tumor progression is a new avenue for cancer research.

*Angiogenesis and lymphangiogenesis*. Angiogenesis, the formation of new blood vessels, is a finely tuned process modulated by stimulatory and inhibitory signals [129,130]. During adulthood, angiogenesis plays a central role in tumor initiation and growth [129]. Vascular endothelial growth factors (VEGFs), angiopoietins (ANGPTs), certain chemokines and hepatocyte growth factor (HGF) are major proangiogenic molecules [131,132]. Lymphangiogenesis is canonically considered pivotal for the diffusion of metastasis to draining lymph nodes [133,134]. Cannabinoids modulate angiogenesis by multiple mechanisms [135]. CBs inhibit angiogenesis in tumors in vivo directly and/or indirectly [136,137,138,139,140]. Cannabinoids suppressed the migration and survival of endothelial cells and the expression of VEGF [141] and the angiogenic features of endothelial cells [119,135]. Various cannabinoids (e.g., CBT, JWH-133) stimulated the release of TIMP-1 from cancer cells [142]. TIMP-1 acted as an endogenous inhibitor of metalloproteinases (MMPs).

Several studies have demonstrated a modulation of immune cells to contribute to cannabinoid antiangiogenic responses. Macrophage-assisted vascular remodeling plays a role in tumor growth [143]. Human lung macrophages (HLMs) produce 2-AG, PEA and OEA and express CB1 and CB2 receptors [29]. Cannabinoid receptor activation by ACEA (CB1 agonist) and JWH-133 (CB2 agonist) inhibited LPS-induced production of VEGF-A and ANGPTs [29]. Interestingly, CB1/CB2 agonists also inhibited the production of the lymphangiogenic factor VEGF-C from HLMs. Although human monocyte-derived macrophages (MDMs) expressed CB1 and CB2 at the mRNA and protein levels, ACEA and JWH-133 did not modify the production of VEGF-A in response to LPS [29]. More recently, we have found that low concentrations of ACEA and JWH-133 inhibited LPS-induced VEGF-A release from human neutrophils [46]. These cannabinoids did not affect the LPS-induced release of CXCL8 and HGF from neutrophils. Importantly, ACEA and JWH-133 inhibited the angiogenic response (i.e., number of tubules and tubule length) induced by LPS-activated neutrophils. Collectively, the results of these studies indicate that CB1 and CB2 receptors are functionally present on two immune cells (i.e., macrophages and neutrophils) present in TME that play a prominent role in tumorigenesis [129,144,145]. 

*Energy metabolism.* Mitochondria produce and release ROS, which play an important role in energy metabolism. During the apoptotic process ROS levels increase [146] by acting on the ERK and ROS pathways, and reduce the expression of Id-1, an inhibitor of basic helix-loop-helix transcription factors and regulator of the proliferative, angiogenic and metastatic processes of various tumors [147,148]. In pancreatic adenocarcinoma, CBs alter the AMP/ATP ratio [101], and associated with the chemotherapeutic gemcitabine, abolish the proliferation of cancer cells through ROS-induced autophagy [102]. Other groups have confirmed the importance of the effects of CBs on tumor energy metabolism in different tumors [149,150]. A recent review extensively examined the role of cannabinoids in experimental and human cancers [64].

**Figure 1:**
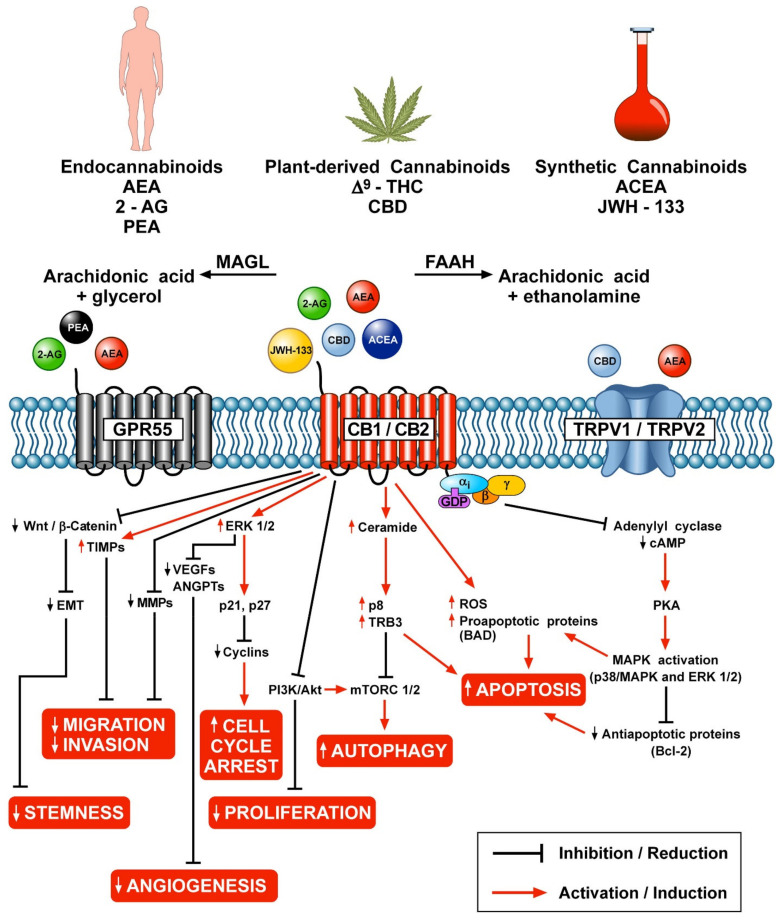
Schematic representation of the potential anticancer effects of endocannabinoids, phytocannabinoids and synthetic cannabinoids through the activation of CB1/CB2, GPR55 and TRPV1/TRPV2 receptors.

Figure 1 schematically illustrates the different mechanisms by which endocannabinoids, phytocannabinoids and synthetic cannabinoids can modulate various aspects of tumor growth through the engagement of CB1/CB2, GPR55 and TRPV1/TRPV2 receptors.

## 4. The Endocannabinoid System as Gate-Keeper of the Immune System

In the 1970s, several groups of investigators started to evaluate the effects of CBs on different immune cells [151]. It has been suggested that the ECS contributes to maintaining the immune homeostasis and functions as a gate-keeper of the immune system [39,152,153,154,155,156] through the activation of classical and non-classical receptors [157,158,159]. Almost all immune cells express and interact with members of the ECS [160]. Human peripheral blood immune cells (i.e., B cells, NK cells, monocytes, neutrophils, eosinophils and CD8^+^ and CD4^+^ lymphocytes) express different degrees of CB receptors [34,70]. The state of cellular activation modulates the different expression of CB receptors. For instance, phorbol 12-myristate 13-acetate (PMA) downregulates the CB1 receptor in T cells whereas it upregulates it in B cells [161], Jurkat T cells and macrophages [162]. 

The tumor microenvironment (TME) contains a plethora of immune cells (i.e., macrophages, neutrophils, mast cells, eosinophils, CD8^+^ and CD4^+^ T cells, etc.) which produce a multitude of mediators that promote cell proliferation, angiogenesis, lymphangiogenesis, and the formation of metastasis [163,164,165]. Although surgery, radiotherapy, chemotherapy, endocrine therapy or immunotherapy are used, alone or in combination, to treat different types of cancer, due to the emergence of drug resistance and relapse, metastasis and death can occur [166,167]. Therefore, it is important to find novel therapeutic approaches. The expression of classical (CB1 and CB2) and non-classical endocannabinoid receptors in immune cells may represent novel targets in the treatment of several tumors [50]. Moreover, there is ample evidence that ECs modulate key functions of immune cells and tumor growth [78,160,168,169,170]. Importantly, cannabinoids can affect tumor growth through the inhibition of angiogenesis and lymphangiogenesis [29,46,171]. Collectively, these findings suggest that selective CB ligands might have a promising potential for the treatment of cancer [64,81]. 

*T lymphocytes.* Different subsets of T cells play fundamental roles in cell-mediated immunity [172,173]. Within the TME, several T cell types are involved in the generation of antitumor immunity [174]. It is not yet clear whether human and mouse T cell subsets produce CBs, but they express both receptors [175,176,177,178,179,180,181]. CB1 receptors, poorly expressed at baseline, are upregulated in cannabinoid-induced T cell polarization [175,176]. CBs regulate the T cell proliferation, reduce their cytolytic activity, and modulate the profile of Th subsets (Th1/Th2) [177,179,180,182]. AEA inhibits human lymphocyte proliferation by mechanisms independent of CB receptors [178], and induces cell death by apoptosis [180]. It was also shown that 2-AG reduced the expression of IL-2 in activated Jurkat T cells [183]. CBs may also be responsible for the conversion of Th1 cells to the Th2 profile, increasing the production of Th2 cytokines (IL-4, IL-5, IL-13) and decreasing those of Th1 type (IL-2, IL-12, and IFN-γ) [177,181]. CD4^+^ T regulatory (Treg) cells potently and specifically inhibit B cell responses and play a role in autoimmune responses and cancer [184,185]. It has been suggested that CBs can up-regulate Foxp3^+^ Tregs and down-regulate inflammatory cytokines [186]. There is overwhelming evidence that CD4^+^ [187,188] and CD8^+^ T cells [189] are highly heterogeneous. The selective distribution of CB1 and CB2 receptors on different subsets of CD4^+^ and CD8^+^ T cells involved in tumor growth is largely unknown. 

*Macrophages.* Macrophages are immune cells resident in all tissues and play a central role in both physiological and pathological processes [190]. Cell line and peritoneal macrophages have been used to study the biosynthesis, uptake and degradation of ECs [191,192,193,194,195]. Mouse and rat macrophages express both CB1 and CB2 receptors [196,197]. There is some evidence that the CB2 receptor is predominantly expressed in cell line macrophages [198,199]. Lypopolisaccharide (LPS) and platelet activating factor (PAF) can induce the synthesis of ECs in mouse macrophages [197,200]. Macrophages express and release AEA, 2-AG, OEA and PEA, which activate CB receptors through different mechanisms [195,197,199,200,201]. CB receptor activation modulates macrophage migration [196,202], antigen presentation and phagocytosis [203], the release of inflammatory mediators (i.e., nitric oxide, TNFα, IL-1, IL-6) and the production of arachidonic acid metabolites [198,200,204]. 2-AG induces a rapid polymerization of actin in various types of inflammatory cells, including macrophages [205]. Cannabidiol exerts anti-tumor effects in breast cancer through the inhibition of macrophage migration induced by cancer cell conditioned medium [111]. Recent evidence demonstrates that MAGL regulates CB2-dependent macrophage activation and cancer progression [206]. We have shown that the activation of both CB1 and CB2 receptors on primary human lung macrophages inhibits the LPS-induced release of angiogenic and lymphangiogenic factors [29]. These results suggest that ECs can modulate macrophage-assisted vascular remodeling in cancer. There is compelling evidence that macrophages constitute a highly heterogeneous cell population localized in different compartments [207,208,209]. Further studies will likely characterize the expression and functional roles of CB1/CB2 receptors in different subsets of macrophages. 

*Neutrophils* (*PMNs*). PMNs are innate immunity cells involved in various acute and chronic inflammatory processes, cardiovascular diseases, infectious diseases, asthma and tumors [46,210,211]. The discovery of CB receptors on PMNs dates back to the 1990s [34]. Their activation mediates the immunosuppressive functions of PMNs [212]. Δ^9^-THC inhibits chemotaxis of human PMNs [213,214]. GPR55 is expressed on human neutrophils and its activation increases the migratory response to 2-AG, inhibiting the degranulation of PMNs and the production of ROS [215]. CB receptor activation also blocks cell differentiation in human myeloid precursor cells [216]. Recent evidence indicates that 2-AG modulates human neutrophils functions, not only through the activation of CB receptors, but also through numerous lipid metabolites [80]. Recently, we have demonstrated that low concentrations of both ACEA (CB1 agonist) and JWH-133 (CB2 agonist) selectively inhibited LPS-induced release of VEGF-A from human neutrophils. In contrast, ACEA and JWH-133 did not affect the release of CXCL8 and hepatocyte growth factor (HGF) from neutrophils. Interestingly, ACEA and JWH-133 inhibited the angiogenic response (i.e., number and length of tubules) induced by LPS-activated neutrophils. These inhibitory effects were presumably due to the reduction of VEGF-A release in neutrophils supernatants. PMNs are among the most common immune cells in TME in different types of human cancers [217,218]. The roles and effects of CBs on PMNs in TME have not yet been defined.

*Mast cells* (*MCs*). MCs are multifunctional immune cells involved in a variety of processes such as allergic inflammation, cancer and defense against parasites [165,219,220]. Rat mast cells express the gene and a functional CB2 receptor [221,222]. Mast cells can have both anti-tumorigenic and pro-tumorigenic effects [223,224]. 2-AG inhibited antigen-induced histamine release from guinea pig mast cells through the engagement of CB2 [225]. It has been shown that CB1 activation of human skin mast cells occurs through the local release of EC [226]. CB1 and CB2 agonists decreased the vascularization of granulomas and the mast cell number and activation [227]. More recently, CB1 was shown to be expressed on human mucosal mast cells [228] and on the connective tissue mast cells of human hair follicles [226]. In the latter study, it was demonstrated that blocking this receptor increased mast cell degranulation without affecting mast cell proliferation in situ [226]. Most of the data obtained in human studies indicate an inhibitory effect of endocannabinoids on mast cell function both directly through CB2 and indirectly through CB1. Both CB1 and CB2 receptors are expressed by mouse bone marrow-derived (BMMCs) mast cells [229]. LPS induced 2-AG from BMMCs through the activation of TLR4. Moreover, TLR4 triggering produced trafficking of CB2 receptors in mast cell vesicles. Finally, 2-AG prevented LPS-induced TNF-α secretion in vivo in a mast cell-dependent model of endotoxemia. It has been shown that AEA inhibits FcεRI-dependent degranulation and cytokine synthesis in mast cells through CB2 and GPR55 receptor activation [230]. The role of CB1 and CB2 receptors in mast cells infiltrating TME in different cancers is largely unknown. However, research in this field is still evolving and it is clear that further investigations are needed to reveal the role of CB receptors in mast cell involvement in tumorigenesis.

*Monocytes.* Monocytes are innate immune cells involved in the maintenance of tissue homeostasis [231] and in pathological processes such as cancer [232]. Activated monocytes may be responsible for tumor progression associated with a negative prognosis [233]. By contrast, there is some evidence that monocytes can inhibit metastasis development [234]. 2-AG promotes the migration of both human monocytic leukemia cells and peripheral blood monocytes, whereas AEA does not induce migration of these cells [235]. Furthermore, 2-AG has been shown to recruit inflammatory cells and induce degradation of the extracellular matrix in a CB2-dependent manner [236]. The CB2 receptor agonist JWH-015 reduced human monocyte migration to CCL2/CCL3 and the IFN-γ-induced ICAM-1 expression [236]. Monocytes are subdivided into three major subsets [CD14^++^ CD16^-^ (classical), CD14^++^ CD16^+^ (intermediate) and CD14^+^ CD16^++^ (non-classical)] [237]. GPR55 was particularly expressed in intermediate and in non-classical monocytes [238]. The distribution of CB1/CB2 receptors on different subsets of human monocytes remains to be investigated.

*Natural killer cells (NK cells).* NK cells are cytotoxic lymphocytes that respond quickly to virally-infected cells and cancer cells [239,240]. NK cells express CB1, CB2 and GPR55, and release large amounts of AEA and 2-AG [39]. Δ^9^ -THC inhibits the cytolytic activity of human and mouse NK cells [241,242,243,244]. 2-AG causes chemotaxis of a NK cell line and of human peripheral blood NK cells, whereas AEA does not induce chemotaxis of these cells [245]. Recently, it has been shown that CB2^-/-^ mice displayed elevated numbers of NK cells in the lung [246]. To date, there is evidence of diversity of peripheral blood human NK cells by single-cell RNA sequencing [247]. The characterization of CB1/CB2 and GPR55 receptors in different subsets of blood NK cells and their role in tumorigenesis remain to be explored. 

*Dendritic cells* (*DCs*). DCs are a diverse group of specialized antigen-presenting cells (APCs) playing a central role in inducing primary immune responses, immunological tolerance and ensuring the regulation of responses of T cells [248]. Although DCs constitute a rare immune cell population within tumors and lymphoid organs, these cells are central to initiation and tumor immunity [249]. Murine bone marrow-derived DCs express both CB1 and CB2 receptors [250]. The endogenous cannabinoid system (i.e., AEA, 2-AG, and PEA) is present in human DCs [251]. LPS stimulated the amount of 2-AG in these cells, which expressed both CB1 and CB2. In vivo administration of 2-AG acted as a chemoattract for DCs [252]. Engagement of both CB1 and CB2 receptors also induced apoptosis of DCs [250,252]. In myeloid and plasmacytoid DCs (pDCs), AEA reduced the release of cytokines (i.e., TNF-α and IFN-α) as well as their ability to induce Th1 and Th17 polarization [253]. The role of infiltrating DCs in tumors is controversial [254,255,256]. The mechanisms by which DCs infiltrate the TME may provide new opportunities for therapeutic intervention for ECs [257]. 

*B lymphocytes.* B-cells in TME can exert an antitumor function [258], mediating tumor cell death by apoptosis [259,260] or by the release of IFN-γ [261]. B-cell infiltration is considered a predictor of good clinical outcome in patients with metastatic melanoma [262]. B-cells are immune cells expressing the highest levels of CB1 and CB2 receptors [263,264]. CB2 is a crucial receptor for B cell differentiation [265] and promotes the conversion of IgM to IgE [39]. In mantle cell lymphoma (MCL), a malignant B-cell lymphoma, and in non-Hodgkin’s lymphoma, CB1 and CB2 receptors are overexpressed [266,267]. In MCL, CBs regulate the proliferative process and induce cell death through [267,268]. Activation of both CB1 and CB2 receptors of MCL cells induces the accumulation of ceramide and apoptosis [269]. In chronic lymphocytic leukemia (CLL), CB receptors are overexpressed compared to healthy B-cells and CB1 could be a novel prognostic marker [270].

*Eosinophils.* Eosinophils are involved in parasitic infections, allergies and cancer [271,272,273]. Eosinophils express only the CB2 receptor [274]. 2-AG induced the migration of eosinophils in eosinophil leukemia cells, and this effect was abolished by a CB2 antagonist [235,275,276]. More recently, it has been confirmed that human and mouse eosinophils selectively express the CB2 receptor [277]. Although the pro- and anti-tumorigenic effects of eosinophils are well known [273,278,279], there are still no studies on the contribution of endocannabinoid-eosinophil interactions in TME.

*Basophils.* Peripheral blood basophils express the full tetrameric (αβγ2) form of the high affinity receptor for IgE (FcεRI) and the heterodimeric receptor for IL-3 [164,280]. In addition, human basophils express a wide spectrum of surface receptors [281]. Activation of human basophils induces the release of proinflammatory mediators (histamine and cysteinyl leukotriene C_4_) and the de novo synthesis of several cytokines (IL-4, IL-13, IL-3, CXCL8) [282,283,284] and angiogenic factors [285]. Although increasing evidence indicates that basophils and their mediators are involved in different cancers [286,287], the presence of ECS in these cells is presently unknown.

Table 1 summarizes some main effects of cannabinoids and CB receptor ligands on cells of innate and adaptive immune system.

**Figure 2:**
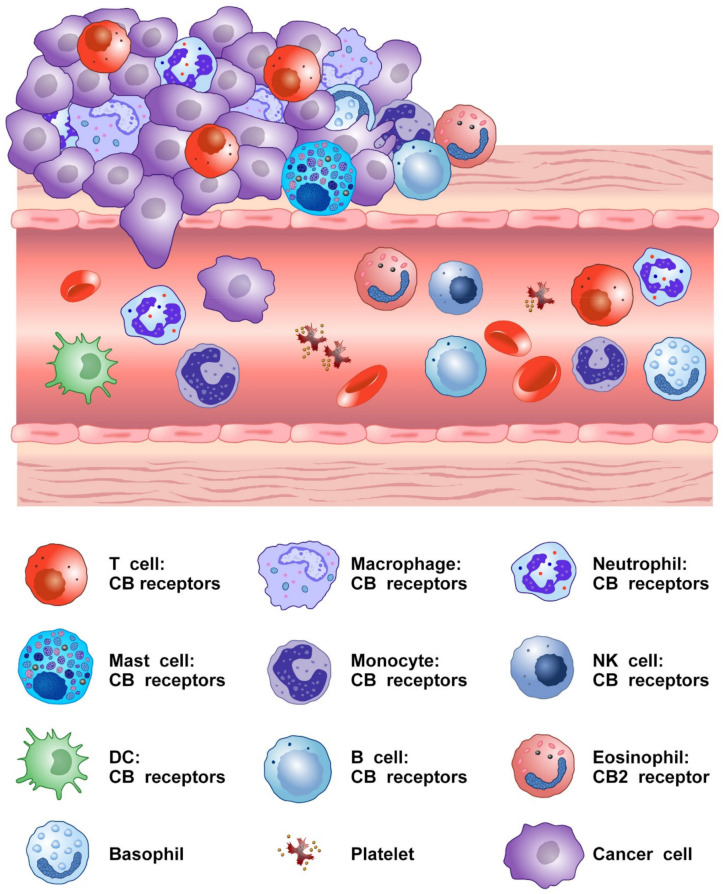
Schematic representation of cells of innate and adaptive immune system expressing CB receptors in the tumor microenvironment.

Figure 2 shows that several cells of innate and adaptive immune system present in TME express CB2 and/or CB1 receptors.

## 5. Conclusions and Future Perspectives

Although the pharmacological properties of *Cannabis sativa* were known in ancient times, the chemical identification and pharmacological properties of its components were only recently characterized [18,63]. There is compelling evidence that the human endocannabinoid system modulates multiple physiological processes mainly through the activation of cannabinoid receptors CB1 and CB2 [8,32,296]. The two receptors share 44% total sequence identity and 68% sequence similarity in the transmembrane regions [11]. However, they differ in their tissue distribution, where CB1 is expressed predominantly in the CNS, whereas CB2 is mainly expressed by several immune cells [18].

Cells of the innate and adaptive immune system play a central role in the initiation and progression of cancer [123,163,190,210,211,286,287]. Cannabinoids can modulate a variety of biological effects which are central to tumor growth [4,81,297,298]. For instance, cannabinoids stimulate cancer cell death, autophagy, inhibit cancer cell proliferation, and activate apoptosis [8,64]. Importantly, the activation of CB receptors inhibits the release of angiogenic and lymphangiogenic factors from human macrophages [29] and neutrophils [46]. Moreover, cannabinoids modulate several biological functions of different immune cells (i.e., T cells, macrophages, monocytes, NK cells, DCs, mast cells, neutrophils, eosinophils) involved in various aspects of cancer initiation and growth [190,299,300]. The expression of CB1 and CB2 on different subsets of immune cells recently identified in TME [301,302] is largely unknown and deserves further studies.

Recently, the structure of the human cannabinoid receptor CB2-G_i_ signaling complex has been characterized [303,304]. These studies have revealed various activation and signaling mechanisms between CB2 and CB1 receptors. These findings could be of paramount importance for the synthesis of specific CB2 and CB1 agonists and antagonists. These compounds will facilitate the characterization of CB receptors on different subsets of immune cells from peripheral blood and in TME. We anticipate that the results that will arise from these studies are likely to aid the rationale for and design of potent and selective CB2 and CB1 ligands with therapeutic potential in cancer.

## Figures and Tables

**Table 1:** Main effects of cannabinoids and CB receptor ligands on cells of innate and adaptive immune system.

Immune Cells	Effects of Cannabinoids and CB Receptor Ligands	Ligands	References
**T Cells** **(human, mouse)**	Inhibition of T cell proliferation	AEA	[180]
	Inhibition of cytolytic activity	Δ^9^-THC	[182]
	Modulation of T_H_1/T_H_2 subsets	Δ^9^-THC	[181]
	Inhibition of IL-2 expression	AEA, 2-AG	[183]
	Inhibition of proliferation and cytokine release	JWH-015, AEA	[179,288]
	Up-regulation of CB receptors	Δ^9^-THC, JWH-015	[177]
	Inhibition of T cell migration	AEA, JWH-133	[289]
**Treg Cells**	Up-regulation of Tregs	Δ^9^-THC	[186]
**Macrophages** **(human, mouse, rat)**	LPS and PAF induce the synthesis of 2-AG		[197,200]
	Inhibition of migration	CP55, 940	[196]
	Inhibition of chemotaxis	O-2137	[202]
	Modulation of phagocytosis	2-AG	[203]
	Inhibition of macrophage cytotoxicity	AEA	[204]
	Inhibition of IL-6 release	Δ^9^-THC, 2-AG	[198]
	Inhibition of TNF-α	2-AG	[290]
	Promotion of ROS production	ACEA	[291]
	Inhibition of angiogenic factor release	ACEA, JWH-133	[29]
	Inhibition of lymphangiogenic factor release	ACEA, JWH-133	[29]
**Neutrophils** **(human)**	Inhibition of ROS production	2-AG	[215]
	Inhibition of chemotaxis	AEA	[51]
	Inhibition of motility	2-AG, JWH-015	[212]
	Inhibition of angiogenic factor release	ACEA, JWH-133	[46]
	Promotion of myeloperoxidase release	2-AG	[292]
	Promotion of LTB_4_ synthesis	2-AG	[292]
**Mast Cells** **(human, guinea pig, mouse)**	Inhibition of histamine release	2-AG, AEA	[225,230]
	Inhibition of activation of skin mast cells	AEA, ACEA	[226]
	Inhibition of TNF-α secretion	2-AG	[229]
	Inhibition of cytokine release	AEA	[230]
	Modulation of angiogenesis	ACEA, JWH-015	[227]
**Monocytes** **(human)**	Promotion of migration	2-AG	[235]
	Inhibition of chemotaxis	JWH-015	[236]
	Inhibition of ICAM-1 expression	JWH-015	[236]
	Inhibition of cytokine release	AEA	[238]
	Modulation of ROS production	AEA, ACEA, WH-015	[291]
**Natural Killer Cells** **(human, mouse)**	Inhibition of cytolytic activity	Δ^9^-THC	[241,242,243,244]
	Promotion of migration	2-AG	[245]
	Modulation of lung NK cells	AM630	[246]
**Dendritic Cells** **(human, mouse)**	Induction of apoptosis	Δ^9^-THC	[250]
	Promotion of chemotaxis	2-AG	[252]
	Inhibition of cytokine release	AEA	[253]
**B cells** **(human)**	B-cell differentiation	2-AG	[265]
	Accumulation of ceramide	Win55	[269]
	Promotion of migration	2-AG	[293,294,295]
**Eosinophils** **(human, mouse)**	Promotion of chemotaxis	2-AG, JWH-133	[275,276,277]

2-AG: 2-arachidonylglycerol; AEA: anandamide; ACEA: arachidonyl-2′-chloroethylamide (CB_1_ agonist); Δ^9^-THC: delta 9-tetrahydrocannabinol; ROS: reactive oxygen species; JWH-015 and JWH-133 are CB2 agonists.

## Data Availability

Not applicable.

## Abbreviations

2-AG	2-arachidonoylglycerol
AEA or anandamide	N-arachidonoylethanolamide
ANGPT	angiopoietin
APC	antigen-presenting cell
B cell	B lymphocyte
BAD	BCL2 associated agonist of cell death
BMMC	bone-marrow-derived mast cell
cAMP	cyclic adenosine monophosphate
CB	cannabinoid
CBD	cannabidiol
Cdk	cyclin-dependent kinase
CLL	chronic lymphocytic leukemia
CNS	central nervous system
COX	cyclooxygenase
CSC	cancer stem cells
CXCR4	C-X-C chemokine receptor type 4
DC	dendritic cell
EC	endocannabinoid
ECS	endocannabinoid system
EGF	epidermal growth factor
EMT	epithelial–mesenchymal transition
ERK	extracellular receptor kinase
FAAH	fatty acid amide hydrolase
GCS	glioblastoma stem-like cells
HGF	hepatocyte growth factor
HLM	human lung macrophage
IFN	interferon
IL	interleukin
LPS	lipopolysaccharide
MAGL	monoacylglycerol lipase
MAPK	mitogen-activated protein kinase
MCL	mantle cell lymphoma
MCs	mast cells
MDM	monocyte-derived macrophage
MMP	matrix metalloproteinase
NK cell	natural killer cell
OEA	oleoylethanolamide
pDC	plasmacytoid dendritic cell
PEA	palmitoylethanolamide
PI3K	phosphoinositide 3-kinase
PKB	pro-tumorigenic protein kinase B
PIGF	placental growth factor
PMN	neutrophil
PPAR	peroxisome proliferator-activated receptors
ROS	reactive oxygen species
SCB	synthetic cannabinoid
T cell	T lymphocyte
Th cell	T helper cell
TIMP	tissue inhibitors of metalloproteinases
TLR	toll-like receptor
TME	tumor microenvironment
TNF	tumor necrosis factor
TRB3	Tribbles homolog 3
TRP	transient receptor potential channels
VEGF	vascular endothelial growth factor
Δ^9^-THC	Δ9-tetrahydrocannabinol.

## References

[1] Jiang H.-E., Li X., Zhao Y.-X., Ferguson D.K., Hueber F., Bera S., Wang Y.-F., Zhao L.-C., Liu C.-J., Li C.-S. (2006). A new insight into Cannabis sativa (Cannabaceae) utilization from 2500-year-old Yanghai Tombs, Xinjiang, China. J. Ethnopharmacol..

[2] Touw M. (1981). The Religious and Medicinal Uses of Cannabis in China, India and Tibet. J. Psychoact. Drugs.

[3] Pisanti S., Bifulco M. (2019). MedicalCannabis: A plurimillennial history of an evergreen. J. Cell. Physiol..

[4] Lal S., Shekher A., Narula A.S., Abrahamse H., Gupta S.C. (2021). Cannabis and its constituents for cancer: History, biogenesis, chemistry and pharmacological activities. Pharmacol. Res..

[5] Robinson S.M., Adinoff B. (2016). The Classification of Substance Use Disorders: Historical, Contextual, and Conceptual Considerations. Behav. Sci..

[6] Zuardi A.W. (2006). History of cannabis as a medicine: A review. Rev. Bras. Psiquiatr..

[7] Pisanti S., Bifulco M. (2017). Modern History of Medical Cannabis: From Widespread Use to Prohibitionism and Back. Trends Pharmacol. Sci..

[8] Bifulco M., Pisanti S. (2015). Pisanti. Medicinal Use of Cannabis in Europe: The Fact That More Countries Legalize the Medicinal Use of Cannabis Should Not Become an Argument for Unfettered and Uncontrolled Use. EMBO Rep..

[9] Ko G.D., Bober S.L., Mindra S., Moreau J.M. (2016). Medical cannabis – the Canadian perspective. J. Pain Res..

[10] Matsuda L.A., Lolait S.J., Brownstein M.J., Young A.C., Bonner T.I. (1990). Structure of a cannabinoid receptor and functional expression of the cloned cDNA. Nat. Cell Biol..

[11] Munro S., Thomas K.L., Abu-Shaar M. (1993). Molecular characterization of a peripheral receptor for cannabinoids. Nat. Cell Biol..

[12] Devane W.A., Hanus L., Breuer A., Pertwee R.G., Stevenson L.A., Griffin G., Gibson D., Mandelbaum A., Etinger A., Mechoulam R. (1992). Isolation and structure of a brain constituent that binds to the cannabinoid receptor. Science.

[13] Mechoulam R., Ben-Shabat S., Hanus L., Ligumsky M., Kaminski N.E., Schatz A.R., Gopher A., Almog S., Martin B.R., Compton D.R. (1995). Identification of an Endogenous 2-Monoglyceride, Present in Canine Gut, That Binds to Canna-binoid Receptors. Biochem. Pharmacol..

[14] Sugiura T., Kondo S., Sukagawa A., Nakane S., Shinoda A., Itoh K., Yamashita A., Waku K. (1995). 2-Arachidonoylglycerol: A Possible Endogenous Cannabinoid Receptor Ligand in Brain. Biochem. Biophys. Res. Commun..

[15] Di Marzo V., Fontana A. (1995). Anandamide, an endogenous cannabinomimetic eicosanoid: ‘Killing two birds with one stone. Prostaglandins Leukot. Essent. Fat. Acids.

[16] Bisogno T., Howell F., Williams G., Minassi A., Cascio M.G., Ligresti A., Matias I., Schiano-Moriello A., Paul P., Williams E.-J. (2003). Cloning of the first sn1-DAG lipases points to the spatial and temporal regulation of endocannabinoid signaling in the brain. J. Cell Biol..

[17] Cravatt B.F., Giang D.K., Mayfield S.P., Boger D.L., Lerner R.A., Gilula N.B. (1996). Molecular characterization of an enzyme that degrades neuromodulatory fatty-acid amides. Nat. Cell Biol..

[18] Cristino L., Bisogno T., Di Marzo V. (2020). Cannabinoids and the expanded endocannabinoid system in neurological disorders. Nat. Rev. Neurol..

[19] Andre C.M., Hausman J.-F., Guerriero G. (2016). Cannabis sativa: The Plant of the Thousand and One Molecules. Front. Plant. Sci..

[20] Mechoulam R., Hanuš L. (2000). A historical overview of chemical research on cannabinoids. Chem. Phys. Lipids.

[21] Boychuk D.G., Goddard G., Mauro G., Orellana M.F. (2015). The effectiveness of cannabinoids in the management of chronic nonmalignant neuropathic pain: A systematic review. J. Oral Facial Pain Headache.

[22] Pomorska D.K., Do-Rego J.-C., Do-Rego J.-L., Zubrzycka M., Janecka A. (2016). Opioid and Cannabinoid System in Food Intake. Curr. Pharm. Des..

[23] Dujourdy L., Besacier F. (2017). A study of cannabis potency in France over a 25 years period (1992–2016). Forensic Sci. Int..

[24] Izzo A.A., Borrelli F., Capasso R., Di Marzo V., Mechoulam R. (2009). Non-psychotropic plant cannabinoids: New therapeutic opportunities from an ancient herb. Trends Pharmacol. Sci..

[25] Stinchcomb A.L., Valiveti S., Hammell D.C., Ramsey D.R. (2004). Human Skin Permeation of Del-ta8-Tetrahydrocannabinol, Cannabidiol and Cannabinol. J. Pharm. Pharmacol..

[26] Mechoulam R., Peters M., Murillo-Rodriguez E., Hanus L.O. (2007). Cannabidiol--Recent Advances. Chem. Biodivers..

[27] Espejo-Porras F., Fernández-Ruiz J., Pertwee R.G., Mechoulam R., García C. (2013). Motor effects of the non-psychotropic phytocannabinoid cannabidiol that are mediated by 5-HT1A receptors. Neuropharmacology.

[28] Rumińska A., Dobrzyń A. (2012). The endocannabinoid system and its role in regulation of metabolism in peripheral tissues. Postępy Biochemii.

[29] Staiano R.I., Loffredo S., Borriello F., Iannotti F.A., Piscitelli F., Orlando P., Secondo A., Granata F., Lepore M.T., Fiorelli A. (2016). Human lung-resident macrophages express CB1 and CB2 receptors whose activation inhibits the release of angiogenic and lymphangiogenic factors. J. Leukoc. Biol..

[30] Yang H.-Y.T., Karoum F., Felder C., Badger H., Wang T.-C.L., Markey S.P. (2008). GC/MS Analysis of Anandamide and Quantification of N-Arachidonoylphosphatidylethanolamides in Various Brain Regions, Spinal Cord, Testis, and Spleen of the Rat. J. Neurochem..

[31] Beltramo M., Stella N., Calignano A., Lin S.Y., Makriyannis A., Piomelli D. (1997). Functional Role of High-Affinity Anandamide Transport, as Revealed by Selective Inhibition. Science.

[32] Di Marzo V., Fontana A., Cadas H., Schinelli S., Cimino G., Schwartz J.-C., Piomelli D. (1994). Formation and inactivation of endogenous cannabinoid anandamide in central neurons. Nat. Cell Biol..

[33] Khan M.I., Sobocinska A.A., Czarnecka A.M., Krol M., Botta B., Szczylik C. (2016). The Therapeutic Aspects of the En-docannabinoid System (Ecs) for Cancer and Their Development: From Nature to Laboratory. Curr. Pharm. Des..

[34] Galiegue S., Mary S., Marchand J., Dussossoy D., Carriere D., Carayon P., Bouaboula M., Shire D., le Fur G., Ca-sellas P. (1995). Expression of Central and Peripheral Cannabinoid Receptors in Human Immune Tissues and Leukocyte Subpop-ulations. Eur. J. Biochem..

[35] Maurya N., Velmurugan B.K. (2018). Therapeutic applications of cannabinoids. Chem. Interactions.

[36] Karwad M.A., Macpherson T., Wang B., Theophilidou E., Sarmad S., Barrett D.A., Larvin M., Wright K.L., Lund J.N., O’Sullivan S.E. (2017). Oleoylethanolamine and Palmitoylethanolamine Modulate Intestinal Permeability in Vitro Via Trpv1 and Pparalpha. FASEB J..

[37] Calignano A., La Rana G., Giuffrida A., Piomelli D. (1998). Control of pain initiation by endogenous cannabinoids. Nat. Cell Biol..

[38] Facci L., Toso R.D., Romanello S., Buriani A., Skaper S.D., Leon A. (1995). Mast cells express a peripheral cannabinoid receptor with differential sensitivity to anandamide and palmitoylethanolamide. Proc. Natl. Acad. Sci. USA.

[39] Chiurchiù V., Battistini L., Maccarrone M. (2015). Endocannabinoid signalling in innate and adaptive immunity. Immunology.

[40] Fezza F., Bari M., Florio R., Talamonti E., Feole M., Maccarrone M. (2014). Endocannabinoids, Related Compounds and Their Metabolic Routes. Molecules.

[41] Castaneto M.S., Gorelick D.A., Desrosiers N.A., Hartman R.L., Pirard S., Huestis M.A. (2014). Synthetic cannabinoids: Epidemiology, pharmacodynamics, and clinical implications. Drug Alcohol Depend..

[42] Walsh K.B., Andersen H.K. (2020). Molecular Pharmacology of Synthetic Cannabinoids: Delineating Cb1 Recep-tor-Mediated Cell Signaling. Int. J. Mol. Sci..

[43] Debruyne D., Le Boisselier R. (2015). Emerging drugs of abuse: Current perspectives on synthetic cannabinoids. Subst. Abus. Rehabil..

[44] Lauritsen K.J., Rosenberg H. (2016). Comparison of Outcome Expectancies for Synthetic Cannabinoids and Botanical Ma-rijuana. Am. J. Drug Alcohol Abuse.

[45] Pacher P., Steffens S., Haskó G., Schindler T.H., Kunos G. (2018). Cardiovascular effects of marijuana and synthetic cannabinoids: The good, the bad, and the ugly. Nat. Rev. Cardiol..

[46] Braile M., Cristinziano L., Marcella S., Varricchi G., Marone G., Modestino L., Ferrara A.L., De Ciuceis A., Scala S., Galdiero M.R. (2021). LPS-mediated neutrophil VEGF-A release is modulated by cannabinoid receptor activation. J. Leukoc. Biol..

[47] Cohen K., Weinstein A. (2018). The Effects of Cannabinoids on Executive Functions: Evidence from Cannabis and Synthetic Cannabinoids—A Systematic Review. Brain Sci..

[48] Lossignol D. (2019). Cannabinoids: A new approach for pain control?. Curr. Opin. Oncol..

[49] Javid F.A., Phillips R.M., Afshinjavid S., Verde R., Ligresti A. (2016). Cannabinoid pharmacology in cancer research: A new hope for cancer patients?. Eur. J. Pharmacol..

[50] Fraguas-Sánchez A.I., Martín-Sabroso C., Torres-Suárez A.I. (2018). Insights into the effects of the endocannabinoid system in cancer: A review. Br. J. Pharmacol..

[51] McHugh D., Tanner C., Mechoulam R., Pertwee R.G., Ross R.A. (2007). Inhibition of Human Neutrophil Chemotaxis by Endogenous Cannabinoids and Phytocannabinoids: Evidence for a Site Distinct from CB1and CB2. Mol. Pharmacol..

[52] Atwood B.K., Mackie K. (2010). CB2: A cannabinoid receptor with an identity crisis. Br. J. Pharmacol..

[53] Pertwee R.G. (2005). Pharmacological Actions of Cannabinoids. Handb. Exp. Pharmacol..

[54] Capozzi A., Mattei V., Martellucci S., Manganelli V., Saccomanni G., Garofalo T., Sorice M., Manera C., Misasi R. (2018). Anti-Proliferative Properties and Proapoptotic Function of New CB2 Selective Cannabinoid Receptor Agonist in Jurkat Leukemia Cells. Int. J. Mol. Science.

[55] Carpi S., Fogli S., Polini B., Montagnani V., Podesta A., Breschi M.C., Romanini A., Stecca B., Nieri P. (2017). Tu-mor-Promoting Effects of Cannabinoid Receptor Type 1 in Human Melanoma Cells. Toxicol. Vitro.

[56] Morales P., Jagerovic N. (2019). Antitumor Cannabinoid Chemotypes: Structural Insights. Front. Pharmacol..

[57] Youssif B.G.M., Mohamed A.M., Osman E.E.A., Abou-Ghadir O.F., Elnaggar D.H., Abdelrahman M.H., Treamblu L., Gomaa H.A.M. (2019). 5-Chlorobenzofuran-2-Carboxamides: From Allosteric Cb1 Modulators to Potential Apoptotic An-titumor Agents. Eur. J. Med. Chem..

[58] Lauckner J.E., Jensen J.B., Chen H.-Y., Lu H.-C., Hille B., Mackie K. (2008). GPR55 is a cannabinoid receptor that increases intracellular calcium and inhibits M current. Proc. Natl. Acad. Sci. USA.

[59] Gangadharan V., Selvaraj D., Kurejova M., Njoo C., Gritsch S., Škoricová D., Horstmann H., Offermanns S., Brown A.J., Kuner T. (2013). A novel biological role for the phospholipid lysophosphatidylinositol in nociceptive sensitization via activation of diverse G-protein signalling pathways in sensory nerves in vivo. Pain.

[60] Pertwee R.G. (2009). Emerging strategies for exploiting cannabinoid receptor agonists as medicines. Br. J. Pharmacol..

[61] Coke C.J., Scarlett K.A., Chetram M.A., Jones K.J., Sandifer B.J., Davis A.S., Marcus A.I., Hinton C.V. (2016). Simul-taneous Activation of Induced Heterodimerization between Cxcr4 Chemokine Receptor and Cannabinoid Receptor 2 (Cb2) Reveals a Mechanism for Regulation of Tumor Progression. J. Biol. Chem..

[62] Moreno E., Cavic M., Krivokuca A., Casadó V., Canela E. (2019). The Endocannabinoid System as a Target in Cancer Diseases: Are We There Yet?. Front. Pharmacol..

[63] Di Marzo V., Piscitelli F. (2015). The Endocannabinoid System and its Modulation by Phytocannabinoids. Neurotherapeutics.

[64] Vecera L., Gabrhelik T., Prasil P., Stourac P. (2020). The role of cannabinoids in the treatment of cancer. Bratisl. Med. J..

[65] Morales P., Reggio P.H., Jagerovic N. (2017). An Overview on Medicinal Chemistry of Synthetic and Natural Derivatives of Cannabidiol. Front. Pharmacol..

[66] De Petrocellis L., Nabissi M., Santoni G., Ligresti A. (2017). Actions and Regulation of Ionotropic Cannabinoid Receptors. Rapid Act. Antidepress..

[67] Gambino G., Rizzo V., Giglia G., Ferraro G., Sardo P. (2020). Cannabinoids, TRPV and nitric oxide: The three ring circus of neuronal excitability. Brain Struct. Funct..

[68] Hinz B., Ramer R. (2019). Anti-tumour actions of cannabinoids. Br. J. Pharmacol..

[69] Cheng H., Yip Y., Lim E., Wahli W., Tan N. (2021). PPARs and Tumor Microenvironment: The Emerging Roles of the Metabolic Master Regulators in Tumor Stromal–Epithelial Crosstalk and Carcinogenesis. Cancers.

[70] Angelina A., Perez-Diego M., Lopez-Abente J., Palomares O. (2020). The Role of Cannabinoids in Allergic Diseases: Colle-gium Internationale Allergologicum (Cia) Update 2020. Int. Arch. Allergy Immunol..

[71] Chiurchiù V., Leuti A., Maccarrone M. (2015). Cannabinoid Signaling and Neuroinflammatory Diseases: A Melting pot for the Regulation of Brain Immune Responses. J. Neuroimmune Pharmacol..

[72] Ramer R., Schwarz R., Hinz B. (2019). Modulation of the Endocannabinoid System as a Potential Anticancer Strategy. Front. Pharmacol..

[73] Cravatt B.F., Lichtman A.H. (2004). The endogenous cannabinoid system and its role in nociceptive behavior. J. Neurobiol..

[74] Maccarrone M. (2017). Metabolism of the Endocannabinoid Anandamide: Open Questions after 25 Years. Front. Mol. Neurosci..

[75] Murataeva N., Straiker A., Mackie K. (2014). Parsing the players: 2-arachidonoylglycerol synthesis and degradation in the CNS. Br. J. Pharmacol..

[76] Schrot R.J., Hubbard J.R. (2016). Cannabinoids: Medical implications. Ann. Med..

[77] Zurier R.B., Burstein S.H. (2016). Cannabinoids, inflammation, and fibrosis. FASEB J..

[78] Pandey R., Mousawy K., Nagarkatti M., Nagarkatti P. (2009). Endocannabinoids and immune regulation. Pharmacol. Res..

[79] Alhouayek M., Muccioli G.G. (2014). COX-2-derived endocannabinoid metabolites as novel inflammatory mediators. Trends Pharmacol. Sci..

[80] Turcotte C., Zarini S., Jean S., Martin C., Murphy R.C., Marsolais D., LaViolette M., Blanchet M.-R., Flamand N. (2017). The Endocannabinoid Metabolite Prostaglandin E2 (PGE2)-Glycerol Inhibits Human Neutrophil Functions: Involvement of Its Hydrolysis into PGE2 and EP Receptors. J. Immunol..

[81] Shah S.A., Gupta A.S., Kumar P. (2021). Emerging role of cannabinoids and synthetic cannabinoid receptor 1/cannabinoid receptor 2 receptor agonists in cancer treatment and chemotherapy-associated cancer management. J. Cancer Res. Ther..

[82] Kalbasi A., Ribas A. (2020). Tumour-intrinsic resistance to immune checkpoint blockade. Nat. Rev. Immunol..

[83] Hart S., Fischer O.M., Ullrich A. (2004). Cannabinoids Induce Cancer Cell Proliferation Via Tumor Necrosis Factor Al-pha-Converting Enzyme (Tace/Adam17)-Mediated Transactivation of the Epidermal Growth Factor Receptor. Cancer Res..

[84] Malfitano A.M., Ciaglia E., Gangemi G., Gazzerro P., Laezza C., Bifulco M. (2011). Update on the endocannabinoid system as an anticancer target. Expert Opin. Ther. Targets.

[85] McKallip R.J., Nagarkatti M., Nagarkatti P.S. (2005). Delta-9-Tetrahydrocannabinol Enhances Breast Cancer Growth and Metastasis by Suppression of the Antitumor Immune Response. J. Immunol..

[86] Sailler S., Schmitz K., Jäger E., Ferreiros N., Wicker S., Zschiebsch K., Pickert G., Geisslinger G., Walter C., Tegeder I. (2014). Regulation of circulating endocannabinoids associated with cancer and metastases in mice and humans. Oncoscience.

[87] Zhu L.X., Sharma S., Stolina M., Gardner B., Roth M.D., Tashkin D.P., Dubinett S.M. (2000). Del-ta-9-Tetrahydrocannabinol Inhibits Antitumor Immunity by a Cb2 Receptor-Mediated, Cytokine-Dependent Pathway. J. Immunol..

[88] Demuth D.G., Molleman A. (2006). Cannabinoid signalling. Life Sci..

[89] Munson A.E., Harris L.S., Friedman M.A., Dewey W.L., Carchman R.A. (1975). Antineoplastic Activity of Cannabinoids2. J. Natl. Cancer Inst..

[90] Schwarz R., Ramer R., Hinz B. (2018). Targeting the endocannabinoid system as a potential anticancer approach. Drug Metab. Rev..

[91] Velasco G., Sanchez C., Guzmán M. (2016). Anticancer Mechanisms of Cannabinoids. Curr. Oncol..

[92] Sarfaraz S., Adhami V.M., Syed D.N., Afaq F., Mukhtar H. (2008). Cannabinoids for Cancer Treatment: Progress and Promise: Figure 1. Cancer Res..

[93] Pacher P., Bátkai S., Kunos G. (2006). The Endocannabinoid System as an Emerging Target of Pharmacotherapy. Pharmacol. Rev..

[94] Velasco G., Sanchez C., Guzmán M. (2012). Towards the use of cannabinoids as antitumour agents. Nat. Rev. Cancer.

[95] Sanchez C., De Ceballos M.L., Del Pulgar T.G., Rueda D., Corbacho C., Velasco G., Galve-Roperh I., Huffman J.W., Cajal S.R.Y., Guzmán M. (2001). Inhibition of glioma growth in vivo by selective activation of the CB(2) cannabinoid receptor. Cancer Res..

[96] Carracedo A., Lorente M., Egia A., Blazquez C., Garcia S., Giroux V., Malicet C., Villuendas R., Gironella M., Gonza-lez-Feria L. (2006). The Stress-Regulated Protein P8 Mediates Canna-binoid-Induced Apoptosis of Tumor Cells. Cancer Cell.

[97] Vara D., Salazar M., Olea-Herrero N., Guzman M., Velasco G., Diaz-Laviada I. (2011). Anti-Tumoral Action of Canna-binoids on Hepatocellular Carcinoma: Role of Ampk-Dependent Activation of Autophagy. Cell Death Differ..

[98] Jia W., Hegde V.L., Singh N.P., Sisco D., Grant S., Nagarkatti M., Nagarkatti P.S. (2006). Del-ta9-Tetrahydrocannabinol-Induced Apoptosis in Jurkat Leukemia T Cells Is Regulated by Translocation of Bad to Mito-chondria. Mol. Cancer Res..

[99] Greenhough A., Patsos H.A., Williams A.C., Paraskeva C. (2007). The Cannabinoid Delta(9)-Tetrahydrocannabinol Inhibits Ras-Mapk and Pi3k-Akt Survival Signalling and Induces Bad-Mediated Apoptosis in Colorectal Cancer Cells. Int. J. Cancer.

[100] McKallip R.J., Jia W., Schlomer J., Warren J.W., Nagarkatti P.S., Nagarkatti M. (2006). Cannabidiol-Induced Apoptosis in Human Leukemia Cells: A Novel Role of Cannabidiol in the Regulation of p22phox and Nox4 Expression. Mol. Pharmacol..

[101] Dando I., Donadelli M., Costanzo C., Pozza E.D., D’Alessandro A., Zolla L., Palmieri M. (2013). Cannabinoids inhibit energetic metabolism and induce AMPK-dependent autophagy in pancreatic cancer cells. Cell Death Dis..

[102] Donadelli M., Dando I., Zaniboni T., Costanzo C., Pozza E.D., Scupoli M.T., Scarpa A., Zappavigna S., Marra M., Abbruzzese A. (2011). Gemcitabine/cannabinoid combination triggers autophagy in pancreatic cancer cells through a ROS-mediated mechanism. Cell Death Dis..

[103] Singh N., Hroudova J., Fisar Z. (2015). Cannabinoid-Induced Changes in the Activity of Electron Transport Chain Com-plexes of Brain Mitochondria. J. Mol. Neurosci..

[104] Tedesco L., Valerio A., Dossena M., Cardile A., Ragni M., Pagano C., Pagotto U., Carruba M.O., Vettor R., Nisoli E. (2010). Cannabinoid Receptor Stimulation Impairs Mitochondrial Biogenesis in Mouse White Adipose Tissue, Muscle, and Liver: The Role of eNOS, p38 MAPK, and AMPK Pathways. Diabetes.

[105] Kim W., Doyle M.E., Liu Z., Lao Q., Shin Y.K., Carlson O.D., Kim H.S., Thomas S., Napora J.K., Lee E.K. (2011). Cannabinoids Inhibit Insulin Receptor Signaling in Pancreatic Beta-Cells. Diabetes.

[106] Liu J., Zhou L., Xiong K., Godlewski G., Mukhopadhyay B., Tam J., Yin S., Gao P., Shan X., Pickel J. (2012). Hepatic Cannabinoid Receptor-1 Mediates Diet-Induced Insulin Resistance via Inhibition of Insulin Signaling and Clearance in Mice. Gastroenterology.

[107] Nabissi M., Morelli M.B., Amantini C., Liberati S., Santoni M., Ricci-Vitiani L., Pallini R., Santoni G. (2015). Cannabidiol Stimulates Aml-1a-Dependent Glial Differentiation and Inhibits Glioma Stem-Like Cells Proliferation by Inducing Au-tophagy in a Trpv2-Dependent Manner. Int. J. Cancer.

[108] Caffarel M.M., Sarrio D., Palacios J., Guzman M., Sanchez C. (2006). Delta9-Tetrahydrocannabinol Inhibits Cell Cycle Progression in Human Breast Cancer Cells through Cdc2 Regulation. Cancer Res..

[109] Caffarel M.M., Moreno-Bueno G., Cerutti C., Palacios J., Guzman M., Mechta-Grigoriou F., Sanchez C. (2008). Jund Is Involved in the Antiproliferative Effect of Delta9-Tetrahydrocannabinol on Human Breast Cancer Cells. Oncogene.

[110] Vara D., Morell C., Rodriguez-Henche N., Diaz-Laviada I. (2013). Involvement of Ppargamma in the Antitumoral Action of Cannabinoids on Hepatocellular Carcinoma. Cell Death Dis..

[111] Elbaz M., Nasser M.W., Ravi J., Wani N.A., Ahirwar D.K., Zhao H., Oghumu S., Satoskar A.R., Shilo K., Carson W.E. (2015). Modulation of the Tumor Microenvironment and Inhibition of Egf/Egfr Pathway: Novel Anti-Tumor Mechanisms of Cannabidiol in Breast Cancer. Mol. Oncol..

[112] Yamaguchi H., Wyckoff J., Condeelis J. (2005). Cell migration in tumors. Curr. Opin. Cell Biol..

[113] Cao Z., Shang B., Zhang G., Miele L., Sarkar F.H., Wang Z., Zhou Q. (2013). Tumor cell-mediated neovascularization and lymphangiogenesis contrive tumor progression and cancer metastasis. Biochim. Biophys. Acta (BBA) Bioenerg..

[114] Hall A. (2009). The Cytoskeleton and Cancer. Cancer Metastasis Rev..

[115] Laezza C., Pisanti S., Malfitano A.M., Bifulco M. (2008). The anandamide analog, Met-F-AEA, controls human breast cancer cell migration via the RHOA/RHO kinase signaling pathway. Endocr. Relat. Cancer.

[116] Ramer R., Bublitz K., Freimuth N., Merkord J., Rohde H., Haustein M., Borchert P., Schmuhl E., Linnebacher M., Hinz B. (2011). Cannabidiol inhibits lung cancer cell invasion and metastasis via intercellular adhesion molecule-1. FASEB J..

[117] Ramer R., Hinz B. (2008). Inhibition of Cancer Cell Invasion by Cannabinoids via Increased Expression of Tissue Inhibitor of Matrix Metalloproteinases-1. J. Natl. Cancer Inst..

[118] Takeda S., Okajima S., Miyoshi H., Yoshida K., Okamoto Y., Okada T., Amamoto T., Watanabe K., Omiecinski C.J., Aramaki H. (2012). Cannabidiolic acid, a major cannabinoid in fiber-type cannabis, is an inhibitor of MDA-MB-231 breast cancer cell migration. Toxicol. Lett..

[119] Thapa D., Lee J.S., Heo S.-W., Lee Y.R., Kang K.W., Kwak M.-K., Choi H.G., Kim J.-A. (2011). Novel hexahydrocannabinol analogs as potential anti-cancer agents inhibit cell proliferation and tumor angiogenesis. Eur. J. Pharmacol..

[120] Muller C., Morales P., Reggio P.H. (2019). Cannabinoid Ligands Targeting TRP Channels. Front. Mol. Neurosci..

[121] Moreno E., Andradas C., Medrano M., Caffarel M.M., Pérez-Gómez E., Blasco-Benito S., Gómez-Cañas M., Pazos M.R., Irving A.J., Lluís C. (2014). Targeting CB2-GPR55 Receptor Heteromers Modulates Cancer Cell Signaling. J. Biol. Chem..

[122] Scarlett K.A., White E.-S.Z., Coke C.J., Carter J.R., Bryant L.K., Hinton C.V. (2018). Agonist-induced CXCR4 and CB2 Heterodimerization Inhibits Gα13/RhoA-mediated Migration. Mol. Cancer Res..

[123] Visciano C., Liotti F., Prevete N., Cali’ G., Franco R., Collina F., De Paulis A., Marone G., Santoro M., Melillo R.M. (2015). Mast cells induce epithelial-to-mesenchymal transition and stem cell features in human thyroid cancer cells through an IL-8–Akt–Slug pathway. Oncogene.

[124] Laezza C., D’Alessandro A., Paladino S., Malfitano A.M., Proto M.C., Gazzerro P., Pisanti S., Santoro A., Ciaglia E., Bifulco M. (2012). Group Endocannabinoid Research Anandamide Inhibits the Wnt/Beta-Catenin Signalling Pathway in Human Breast Cancer Mda Mb 231 Cells. Eur. J. Cancer.

[125] Fiore D., Ramesh P., Proto M.C., Piscopo C., Franceschelli S., Anzelmo S., Medema J.P., Bifulco M., Gazzerro P. (2018). Rimonabant Kills Colon Cancer Stem Cells without Inducing Toxicity in Normal Colon Organoids. Front. Pharmacol..

[126] Proto M.C., Fiore D., Piscopo C., Franceschelli S., Bizzarro V., Laezza C., Lauro G., Feoli A., Tosco A., Bifulco G. (2017). Inhibition of Wnt/Beta-Catenin Pathway and Histone Acetyltransferase Activity by Rimonabant: A Therapeutic Target for Colon Cancer. Sci. Rep..

[127] Gustafsson S.B., Wallenius A., Zackrisson H., Popova D., Forshell L.P., Jacobsson S.O.P. (2013). Effects of cannabinoids and related fatty acids upon the viability of P19 embryonal carcinoma cells. Arch. Toxicol..

[128] Aguado T., Carracedo A., Julien B., Velasco G., Milman G., Mechoulam R., Alvarez L., Guzmán M., Galve-Roperh I. (2007). Cannabinoids Induce Glioma Stem-like Cell Differentiation and Inhibit Gliomagenesis. J. Biol. Chem..

[129] De Palma M., Biziato D., Petrova T.V. (2017). Microenvironmental regulation of tumour angiogenesis. Nat. Rev. Cancer.

[130] Varricchi G., Loffredo S., Borriello F., Pecoraro A., Rivellese F., Genovese A., Spadaro G., Marone G. (2019). Superantigenic Activation of Human Cardiac Mast Cells. Int. J. Mol. Sci..

[131] Saharinen P., Leppänen V.-M., Alitalo K. (2017). SnapShot: Angiopoietins and Their Functions. Cell.

[132] Loffredo S., Staiano R.I., Granata F., Genovese A., Marone G. (2013). Immune Cells as a Source and Target of Angiogenic and Lymphangiogenic Factors. Superantigens Superallerg..

[133] Varricchi G., De Paulis A., Marone G., Galli S.J., Paulis D. (2019). Future Needs in Mast Cell Biology. Int. J. Mol. Sci..

[134] Zheng W., Aspelund A., Alitalo K. (2014). Lymphangiogenic factors, mechanisms, and applications. J. Clin. Investig..

[135] Solinas M., Massi P., Cantelmo A., Cattaneo M., Cammarota R., Bartolini D., Cinquina V., Valenti M., Vicentini L., Noonan D. (2012). Cannabidiol inhibits angiogenesis by multiple mechanisms. Br. J. Pharmacol..

[136] Pisanti S., Borselli C., Oliviero O., Laezza C., Gazzerro P., Bifulco M. (2007). Antiangiogenic activity of the endocannabinoid anandamide: Correlation to its tumor-suppressor efficacy. J. Cell. Physiol..

[137] Portella G., Laezza C., Laccetti P., de Petrocellis L., di Marzo V., Bifulco M. (2003). Inhibitory Effects of Cannabinoid Cb1 Receptor Stimulation on Tumor Growth and Metastatic Spreading: Actions on Signals Involved in Angiogenesis and Me-tastasis. FASEB J..

[138] Blázquez C., González-Feria L., Álvarez L., Haro A., Casanova M.L., Guzmán M. (2004). Cannabinoids Inhibit the Vascular Endothelial Growth Factor Pathway in Gliomas. Cancer Res..

[139] Ramer R., Hinz B. (2016). Antitumorigenic targets of cannabinoids–current status and implications. Expert Opin. Ther. Targets.

[140] Ramer R., Hinz B. (2017). Cannabinoids as Anticancer Drugs. Rapid Act. Antidepress..

[141] Blázquez C., Casanova M.L., Planas A., Del Pulgar T.G., Villanueva C., Fernández-Aceñero M.J., Aragonés J., Huffman J.W., Jorcano J.L., Guzmán M. (2003). Inhibition of tumor angiogenesis by cannabinoids. FASEB J..

[142] Ramer R., Fischer S., Haustein M., Manda K., Hinz B. (2014). Cannabinoids inhibit angiogenic capacities of endothelial cells via release of tissue inhibitor of matrix metalloproteinases-1 from lung cancer cells. Biochem. Pharmacol..

[143] Sica A., Mantovani A. (2012). Macrophage plasticity and polarization: In vivo veritas. J. Clin. Investig..

[144] Galdiero M.R., Varricchi G., Loffredo S., Bellevicine C., Lansione T., Ferrara A.L., Iannone R., Di Somma S., Borriello F., Clery E. (2018). Potential involvement of neutrophils in human thyroid cancer. PLoS ONE.

[145] Loffredo S., Borriello F., Iannone R., Ferrara A.L., Galdiero M.R., Gigantino V., Esposito P., Varricchi G., Lambeau G., Cassatella M.A. (2017). Group V Secreted Phospholipase A2 Induces the Release of Proangiogenic and Antiangiogenic Factors by Human Neutrophils. Front. Immunol..

[146] Wallace D.C. (2012). Mitochondria and cancer. Nat. Rev. Cancer.

[147] McAllister S.D., Christian R.T., Horowitz M.P., Garcia A., Desprez P.-Y. (2007). Cannabidiol as a novel inhibitor of Id-1 gene expression in aggressive breast cancer cells. Mol. Cancer Ther..

[148] McAllister S.D., Murase R., Christian R.T., Lau D., Zielinski A.J., Allison J., Almanza C., Pakdel A., Lee J., Limbad C. (2010). Pathways mediating the effects of cannabidiol on the reduction of breast cancer cell proliferation, invasion, and metastasis. Breast Cancer Res. Treat..

[149] Gurley S.N., Abidi A.H., Allison P., Guan P., Duntsch C., Robertson J.H., Kosanke S.D., Keir S.T., Bigner D.D., Elberger A.J. (2012). Mechanism of anti-glioma activity and in vivo efficacy of the cannabinoid ligand KM-233. J. Neuro Oncol..

[150] Whyte D.A., Al-Hammadi S., Balhaj G., Brown O.M., Penefsky H.S., Souid A.-K. (2010). Cannabinoids Inhibit Cellular Respiration of Human Oral Cancer Cells. Pharmacology.

[151] E Mann P., Cohen A.B., Finley T.N., Ladman A.J. (1971). Alveolar macrophages. Structural and functional differences between nonsmokers and smokers of marijuana and tobacco. Lab. Investig..

[152] Chiurchiù V. (2016). Endocannabinoids and Immunity. Cannabis Cannabinoid Res..

[153] Lu Y., Anderson H.D. (2017). Cannabinoid signaling in health and disease. Can. J. Physiol. Pharmacol..

[154] Maccarrone M., Bab I., Bíró T., Cabral G.A., Dey S.K., Di Marzo V., Konje J.C., Kunos G., Mechoulam R., Pacher P. (2015). Endocannabinoid signaling at the periphery: 50 years after THC. Trends Pharmacol. Sci..

[155] Oláh A., Bíró T. (2017). Targeting Cutaneous Cannabinoid Signaling in Inflammation - A “High”-way to Heal?. EBioMedicine.

[156] Zhou J., Burkovskiy I., Yang H., Sardinha J., Lehmann C. (2016). CB2 and GPR55 Receptors as Therapeutic Targets for Systemic Immune Dysregulation. Front. Pharmacol..

[157] O’Sullivan S., Kendall D. (2010). Cannabinoid activation of peroxisome proliferator-activated receptors: Potential for modulation of inflammatory disease. Immunobiology.

[158] Parenti A., De Logu F., Geppetti P., Benemei S. (2016). What is the evidence for the role of TRP channels in inflammatory and immune cells?. Br. J. Pharmacol..

[159] Santoni G., Cardinali C., Morelli M.B., Santoni M., Nabissi M., Amantini C. (2015). Danger- and pathogen-associated molecular patterns recognition by pattern-recognition receptors and ion channels of the transient receptor potential family triggers the inflammasome activation in immune cells and sensory neurons. J. Neuroinflamm..

[160] Turcotte C., Chouinard F., Lefebvre J.S., Flamand N. (2015). Regulation of Inflammation by Cannabinoids, the Endocan-nabinoids 2-Arachidonoyl-Glycerol and Arachidonoyl-Ethanolamide, and Their Metabolites. J. Leukoc. Biol..

[161] Noe S.N., Newton C., Widen R., Friedman H., Klein T.W. (2000). Anti-CD40, anti-CD3, and IL-2 stimulation induce contrasting changes in CB1 mRNA expression in mouse splenocytes. J. Neuroimmunol..

[162] Daaka Y., Friedman H., Klein T.W. (1996). Cannabinoid receptor proteins are increased in Jurkat, human T-cell line after mitogen activation. J. Pharmacol. Exp. Ther..

[163] Cristinziano L., Modestino L., Loffredo S., Varricchi G., Braile M., Ferrara A.L., De Paulis A., Antonelli A., Marone G., Galdiero M.R. (2020). Anaplastic Thyroid Cancer Cells Induce the Release of Mitochondrial Extracellular DNA Traps by Viable Neutrophils. J. Immunol..

[164] Ferrari S.M., Fallahi P., Galdiero M.R., Ruffilli I., Elia G., Ragusa F., Paparo S.R., Patrizio A., Mazzi V., Varricchi G. (2019). Immune and Inflammatory Cells in Thyroid Cancer Microenvironment. Int. J. Mol. Sci..

[165] Sammarco G., Varricchi G., Ferraro V., Ammendola M., De Fazio M., Altomare D.F., Luposella M., Maltese L., Currò G., Marone G. (2019). Mast Cells, Angiogenesis and Lymphangiogenesis in Human Gastric Cancer. Int. J. Mol. Sci..

[166] Binabaj M.M., Bahrami A., ShahidSales S., Joodi M., Mashhad M.J., Hassanian S.M., Anvari K., Avan A. (2018). The prognostic value of MGMT promoter methylation in glioblastoma: A meta-analysis of clinical trials. J. Cell. Physiol..

[167] Tajbakhsh A., Mokhtari-Zaer A., Rezaee M., Afzaljavan F., Rivandi M., Hassanian S.M., Ferns G.A., Pasdar A., Avan A. (2017). Therapeutic Potentials of BDNF/TrkB in Breast Cancer; Current Status and Perspectives. J. Cell. Biochem..

[168] Klein T.W., Newton C.A., Friedman H. (2001). Cannabinoids and the Immune System. Pain Res. Manag..

[169] Śledziński P., Zeyland J., Słomski R., Nowak A.N.-T. (2018). The current state and future perspectives of cannabinoids in cancer biology. Cancer Med..

[170] Tanasescu R., Constantinescu C.S. (2010). Cannabinoids and the immune system: An overview. Immunobiology.

[171] Casanova M.L., Blázquez C., Martínez-Palacio J., Villanueva C., Fernández-Aceñero M.J., Huffman J.W., Jorcano J.L., Guzmán M. (2003). Inhibition of skin tumor growth and angiogenesis in vivo by activation of cannabinoid receptors. J. Clin. Investig..

[172] Sharif H., Acharya S., Dhondalay G.K.R., Varricchi G., Krasner-Macleod S., Laisuan W., Switzer A., Lenormand M., Kashe E., Parkin R.V. (2021). Altered chromatin landscape in circulating T follicular helper and regulatory cells following grass pollen subcutaneous and sublingual immunotherapy. J. Allergy Clin. Immunol..

[173] Varricchi G., Pecoraro A., Marone G., Criscuolo G., Spadaro G., Genovese A., Marone G. (2018). Thymic Stromal Lymphopoietin Isoforms, Inflammatory Disorders, and Cancer. Front. Immunol..

[174] Garner H., de Visser K.E. (2020). Immune Crosstalk in Cancer Progression and Metastatic Spread: A Complex Conversa-tion. Nat. Rev. Immunol..

[175] Börner C., Bedini A., Höllt V., Kraus J. (2007). Analysis of Promoter Regions Regulating Basal and Interleukin-4-Inducible Expression of the Human CB1 Receptor Gene in T Lymphocytes. Mol. Pharmacol..

[176] Börner C., Höllt V., Kraus J. (2007). Activation of Human T Cells Induces Upregulation of Cannabinoid Receptor Type 1 Transcription. Neuroimmunomodulation.

[177] Börner C., Smida M., Höllt V., Schraven B., Kraus J. (2009). Cannabinoid Receptor Type 1- and 2-mediated Increase in Cyclic AMP Inhibits T Cell Receptor-triggered Signaling. J. Biol. Chem..

[178] Derocq J.M., Bouaboula M., Marchand J., Rinaldi-Carmona M., Segui M., Casellas P. (1998). The Endogenous Cannabinoid Anandamide Is a Lipid Messenger Activating Cell Growth Via a Cannabinoid Receptor-Independent Pathway in Hema-topoietic Cell Lines. FEBS Lett..

[179] Lombard C., Nagarkatti M., Nagarkatti P. (2007). CB2 cannabinoid receptor agonist, JWH-015, triggers apoptosis in immune cells: Potential role for CB2-selective ligands as immunosuppressive agents. Clin. Immunol..

[180] Schwarz H., Blanco F.J., Lotz M. (1994). Anadamide, an endogenous cannabinoid receptor agonist inhibits lymphocyte proliferation and induces apoptosis. J. Neuroimmunol..

[181] Yuan M., Kiertscher S.M., Cheng Q., Zoumalan R., Tashkin D.P., Roth M.D. (2002). Delta 9-Tetrahydrocannabinol Reg-ulates Th1/Th2 Cytokine Balance in Activated Human T Cells. J. Neuroimmunol..

[182] Fischer-Stenger K., Updegrove A.W., Cabral G.A. (1992). Delta 9-Tetrahydrocannabinol Decreases Cytotoxic T Lympho-cyte Activity to Herpes Simplex Virus Type 1-Infected Cells. Proc. Soc. Exp. Biol. Med..

[183] Rockwell C.E., Raman P., Kaplan B., Kaminski N.E. (2008). A COX-2 metabolite of the endogenous cannabinoid, 2-arachidonyl glycerol, mediates suppression of IL-2 secretion in activated Jurkat T cells. Biochem. Pharmacol..

[184] Matarese G., De Rosa V., La Cava A. (2008). Regulatory CD4 T cells: Sensing the environment. Trends Immunol..

[185] Colamatteo A., Carbone F., Bruzzaniti S., Galgani M., Fusco C., Maniscalco G.T., Di Rella F., De Candia P., De Rosa V. (2020). Molecular Mechanisms Controlling Foxp3 Expression in Health and Autoimmunity: From Epigenetic to Post-translational Regulation. Front. Immunol..

[186] Hegde V.L., Hegde S., Cravatt B.F., Hofseth L.J., Nagarkatti M., Nagarkatti P.S. (2008). Attenuation of Experimental Autoimmune Hepatitis by Exogenous and Endogenous Cannabinoids: Involvement of Regulatory T Cells. Mol. Pharmacol..

[187] Gowthaman U., Chen J.S., Zhang B., Flynn W.F., Lu Y., Song W., Joseph J., Gertie J.A., Xu L., Collet M.A. (2019). Identifi-cation of a T Follicular Helper Cell Subset That Drives Anaphylactic Ige. Science.

[188] Zemmour D., Kiner E., Benoist C. (2020). CD4+ teff cell heterogeneity: The perspective from single-cell transcriptomics. Curr. Opin. Immunol..

[189] Thommen D.S., Koelzer V.H., Herzig P., Roller A., Trefny M., Dimeloe S., Kiialainen A., Hanhart J., Schill C., Hess C. (2018). A transcriptionally and functionally distinct PD-1+ CD8+ T cell pool with predictive potential in non-small-cell lung cancer treated with PD-1 blockade. Nat. Med..

[190] Locati M., Curtale G., Mantovani A. (2020). Diversity, Mechanisms, and Significance of Macrophage Plasticity. Annu. Rev. Pathol. Mech. Dis..

[191] Bisogno T., Maurelli S., Melck D., de Petrocellis L., di Marzo V. (1997). Biosynthesis, Uptake, and Degradation of Anan-damide and Palmitoylethanolamide in Leukocytes. J. Biol. Chem..

[192] Di Marzo V., De Petrocellis L., Sepe N., Buono A. (1996). Biosynthesis of anandamide and related acylethanolamides in mouse J774 macrophages and N18 neuroblastoma cells. Biochem. J..

[193] Kuwae T., Shiota Y., Schmid P.C., Krebsbach R., Schmid H.H. (1999). Biosynthesis and turnover of anandamide and otherN-acylethanolamines in peritoneal macrophages. FEBS Lett..

[194] Schmid P.C., Kuwae T., Krebsbach R.J., Schmid H.H. (1997). Anandamide and other N-acylethanolamines in mouse peritoneal macrophages. Chem. Phys. Lipids.

[195] Hsu K.L., Tsuboi K., Adibekian A., Pugh H., Masuda K., Cravatt B.F. (2012). Daglbeta Inhibition Perturbs a Lipid Network Involved in Macrophage Inflammatory Responses. Nat. Chem. Biol..

[196] Sacerdote P., Massi P., Panerai A.E., Parolaro D. (2000). In vivo and in vitro treatment with the synthetic cannabinoid CP55,940 decreases the in vitro migration of macrophages in the rat: Involvement of both CB1 and CB2 receptors. J. Neuroimmunol..

[197] Di Marzo V., Bisogno T., De Petrocellis L., Melck D., Orlando P., Wagner J.A., Kunos G. (1999). Biosynthesis and inactivation of the endocannabinoid 2-arachidonoylglycerol in circulating and tumoral macrophages. JBIC J. Biol. Inorg. Chem..

[198] Chang Y.-H., Lee S.T., Lin W.-W. (2001). Effects of cannabinoids on LPS-stimulated inflammatory mediator release from macrophages: Involvement of eicosanoids. J. Cell. Biochem..

[199] Ross R.A., Brockie H.C., Pertwee R.G. (2000). Inhibition of Nitric Oxide Production in Raw264.7 Macrophages by Can-nabinoids and Palmitoylethanolamide. Eur. J. Pharmacol..

[200] Berdyshev E.V., Schmid P.C., Krebsbach R.J., Schmid H.H.O. (2001). Activation of PAF receptors results in enhanced synthesis of 2-arachidonoylglycerol (2-AG) in immune cells. FASEB J..

[201] Massi P., Vaccani A., Bianchessi S., Costa B., Macchi P., Parolaro D. (2006). The non-psychoactive cannabidiol triggers caspase activation and oxidative stress in human glioma cells. Cell. Mol. Life Sci..

[202] Raborn E.S., Marciano-Cabral F., Buckley N.E., Martin B.R., Cabral G.A. (2008). The Cannabinoid Del-ta-9-Tetrahydrocannabinol Mediates Inhibition of Macrophage Chemotaxis to Rantes/Ccl5: Linkage to the Cb2 Receptor. J. Neuroimmun. Pharmacol..

[203] Gokoh M., Kishimoto S., Oka S., Sugiura T. (2007). 2-Arachidonoylglycerol Enhances the Phagocytosis of Opsonized Zy-mosan by Hl-60 Cells Differentiated into Macrophage-Like Cells. Biol. Pharm. Bull..

[204] Cabral G.A., Toney D.M., Fischer-Stenger K., Harrison M.P., Marciano-Cabral F. (1995). Anandamide Inhibits Macro-phage-Mediated Killing of Tumor Necrosis Factor-Sensitive Cells. Life Sci..

[205] Gokoh M., Kishimoto S., Oka S., Mori M., Waku K., Ishima Y., Sugiura T. (2005). 2-Arachidonoylglycerol, an endogenous cannabinoid receptor ligand, induces rapid actin polymerization in HL-60 cells differentiated into macrophage-like cells. Biochem. J..

[206] Xiang W., Shi R., Kang X., Zhang X., Chen P., Zhang L., Hou A., Wang R., Zhao Y., Zhao K. (2018). Monoacylglycerol Lipase Regulates Can-nabinoid Receptor 2-Dependent Macrophage Activation and Cancer Progression. Nat. Commun..

[207] Ginhoux F., Guilliams M. (2016). Tissue-Resident Macrophage Ontogeny and Homeostasis. Immunity.

[208] Gordon S., Plüddemann A. (2017). Tissue macrophages: Heterogeneity and functions. BMC Biol..

[209] Chakarov S., Lim H.Y., Tan L., Lim S.Y., See P., Lum J., Zhang X.-M., Foo S., Nakamizo S., Duan K. (2019). Two distinct interstitial macrophage populations coexist across tissues in specific subtissular niches. Science.

[210] Galdiero M.R., Bianchi P., Grizzi F., di Caro G., Basso G., Ponzetta A., Bonavita E., Barbagallo M., Tartari S., Polen-tarutti N. (2016). Occurrence and Signif-icance of Tumor-Associated Neutrophils in Patients with Colorectal Cancer. Int. J. Cancer.

[211] Galdiero M.R., Varricchi G., Marone G. (2016). The immune network in thyroid cancer. OncoImmunology.

[212] Kurihara R., Tohyama Y., Matsusaka S., Naruse H., Kinoshita E., Tsujioka T., Katsumata Y., Yamamura H. (2006). Effects of Peripheral Cannabinoid Receptor Ligands on Motility and Polarization in Neutrophil-Like Hl60 Cells and Human Neu-trophils. J. Biol. Chem..

[213] Coffelt S.B., Wellenstein M.D., De Visser K.E. (2016). Neutrophils in cancer: Neutral no more. Nat. Rev. Cancer.

[214] Deusch E., Kraft B., Nahlik G., Weigl L., Hohenegger M., Kress H.G. (2003). No evidence for direct modulatory effects of delta 9-tetrahydrocannabinol on human polymorphonuclear leukocytes. J. Neuroimmunol..

[215] Balenga N.A., Aflaki E., Kargl J., Platzer W., Schroder R., Blattermann S., Kostenis E., Brown A.J., Heinemann A., Waldhoer M. (2011). Gpr55 Regulates Cannabinoid 2 Receptor-Mediated Responses in Human Neutrophils. Cell Res..

[216] Jordà M.A., Löwenberg B., Delwel R. (2003). The peripheral cannabinoid receptor Cb2, a novel oncoprotein, induces a reversible block in neutrophilic differentiation. Blood.

[217] Kargl J., Busch S.E., Yang G.H.Y., Kim K.-H., Hanke M.L., Metz H.E., Hubbard J.J., Lee S.M., Madtes D.K., McIntosh M.W. (2017). Neutrophils dominate the immune cell composition in non-small cell lung cancer. Nat. Commun..

[218] Shaul M.E., Fridlender Z.G. (2019). Tumour-associated neutrophils in patients with cancer. Nat. Rev. Clin. Oncol..

[219] Borriello F., Granata F., Varricchi G., Genovese A., Triggiani M., Marone G. (2014). Immunopharmacological modulation of mast cells. Curr. Opin. Pharmacol..

[220] Metcalfe D.D., Baram D., Mekori Y.A. (1997). Mast cells. Physiol. Rev..

[221] Lau A.H.Y., Chow S.S.M. (2003). Effects of cannabinoid receptor agonists on immunologically induced histamine release from rat peritoneal mast cells. Eur. J. Pharmacol..

[222] Samson M.-T., Small-Howard A., Shimoda L.M.N., Koblan-Huberson M., Stokes A.J., Turner H. (2003). Differential Roles of CB1 and CB2 Cannabinoid Receptors in Mast Cells. J. Immunol..

[223] Siebenhaar F., Redegeld F.A., Bischoff S.C., Gibbs B.F., Maurer M. (2018). Mast Cells as Drivers of Disease and Therapeutic Targets. Trends Immunol..

[224] Varricchi G., Galdiero M.R., Loffredo S., Marone G., Iannone R., Marone G., Granata F. (2017). Are Mast Cells MASTers in Cancer?. Front. Immunol..

[225] Vannacci A., Giannini L., Passani M.B., Di Felice A., Pierpaoli S., Zagli G., Fantappiè O., Mazzanti R., Masini E., Mannaioni P.F. (2004). The Endocannabinoid 2-Arachidonylglycerol Decreases the Immunological Activation of Guinea Pig Mast Cells: Involvement of Nitric Oxide and Eicosanoids. J. Pharmacol. Exp. Ther..

[226] Sugawara K., Bíró T., Tsuruta D., Tóth B.I., Kromminga A., Zákány N., Zimmer A., Funk W., Gibbs B.F., Zimmer A. (2012). Endocannabinoids limit excessive mast cell maturation and activation in human skin. J. Allergy Clin. Immunol..

[227] De Filippis D., Russo A., D’Amico A., Esposito G., Pietropaolo C., Cinelli M., Russo G., Iuvone T. (2008). Cannabinoids Reduce Granuloma-Associated Angiogenesis in Rats by Controlling Transcription and Expression of Mast Cell Protease-5. Br. J. Pharmacol..

[228] Sugawara K., Zakany N., Hundt T., Emelianov V., Tsuruta D., Schafer C., Kloepper J.E., Biro T., Paus R. (2013). Canna-binoid Receptor 1 Controls Human Mucosal-Type Mast Cell Degranulation and Maturation in Situ. J. Allergy Clin. Immunol..

[229] Espinosa-Riquer Z.P., Ibarra-Sanchez A., Vibhushan S., Bratti M., Charles N., Blank U., Rodriguez-Manzo G., Gonzalez-Espinosa C. (2019). Tlr4 Receptor Induces 2-Ag-Dependent Tolerance to Lipopolysaccharide and Trafficking of Cb2 Re-ceptor in Mast Cells. J. Immunol..

[230] Cruz S.L., Sanchez-Miranda E., Castillo-Arellano J.I., Cervantes-Villagrana R.D., Ibarra-Sanchez A., Gonza-lez-Espinosa C. (2018). Anandamide Inhibits Fcepsilonri-Dependent Degranulation and Cytokine Synthesis in Mast Cells through Cb2 and Gpr55 Receptor Activation. Possible Involvement of Cb2-Gpr55 Heteromers. Int. Immunopharmacol..

[231] Guilliams M., Mildner A., Yona S. (2018). Developmental and Functional Heterogeneity of Monocytes. Immunity.

[232] Canè S., Ugel S., Trovato R., Marigo I., De Sanctis F., Sartoris S., Bronte V. (2019). The Endless Saga of Monocyte Diversity. Front. Immunol..

[233] Huang S.H., Waldron J.N., Milosevic M., Shen X., Ringash J., Su J., Tong L., Perez-Ordonez B., Weinreb I., Bayley A.J. (2014). Prognostic value of pretreatment circulating neutrophils, monocytes, and lymphocytes in oropharyngeal cancer stratified by human papillomavirus status. Cancer.

[234] Hanna R.N., Cekic C., Sag D., Tacke R., Thomas G.D., Nowyhed H., Herrley E., Rasquinha N., McArdle S., Wu R. (2015). Patrolling monocytes control tumor metastasis to the lung. Science.

[235] Kishimoto S., Gokoh M., Oka S., Muramatsu M., Kajiwara T., Waku K., Sugiura T. (2003). 2-Arachidonoylglycerol Induces the Migration of HL-60 Cells Differentiated into Macrophage-like Cells and Human Peripheral Blood Monocytes through the Cannabinoid CB2 Receptor-dependent Mechanism. J. Biol. Chem..

[236] Montecucco F., Burger F., Mach F., Steffens S. (2008). CB2 cannabinoid receptor agonist JWH-015 modulates human monocyte migration through defined intracellular signaling pathways. Am. J. Physiol. Circ. Physiol..

[237] Shi C., Pamer E.G. (2011). Monocyte recruitment during infection and inflammation. Nat. Rev. Immunol..

[238] Chiurchiu V., Lanuti M., de Bardi M., Battistini L., Maccarrone M. (2015). The Differential Characterization of Gpr55 Re-ceptor in Human Peripheral Blood Reveals a Distinctive Expression in Monocytes and Nk Cells and a Proinflammatory Role in These Innate Cells. Int. Immunol..

[239] Mace E.M., Orange J.S. (2018). Emerging insights into human health and NK cell biology from the study of NK cell deficiencies. Immunol. Rev..

[240] Chiossone L., Dumas P.-Y., Vienne M., Vivier E. (2018). Natural killer cells and other innate lymphoid cells in cancer. Nat. Rev. Immunol..

[241] Klein T.W., Newton C., Friedman H. (1987). Inhibition of natural killer cell function by Marijuana components. J. Toxicol. Environ. Heal. Part. A.

[242] Patel V., Borysenko M., Kumar M., Millard W. (1986). Effects of acute and subchronic Delta 9-tetrahydrocannabinol administration on the plasma catecholamine, beta-endorphin, and corticosterone levels and splenic natural killer cell activity in rats. J. Ethnopharmacol..

[243] Specter S.C., Klein T.W., Newton C., Mondragon M., Widen R., Friedman H. (1986). Marijuana effects on immunity: Suppression of human natural killer cell activity by delta-9-tetrahydrocannabinol. Int. J. Immunopharmacol..

[244] Massi P., Fuzio D., Viganò D., Sacerdote P., Parolaro D. (2000). Relative involvement of cannabinoid CB(1) and CB(2) receptors in the Delta(9)-tetrahydrocannabinol-induced inhibition of natural killer activity. Eur. J. Pharmacol..

[245] Kishimoto S., Muramatsu M., Gokoh M., Oka S., Waku K., Sugiura T. (2005). Endogenous Cannabinoid Receptor Ligand Induces the Migration of Human Natural Killer Cells. J. Biochem..

[246] Ferrini M.E., Hong S., Stierle A., Stella N., Roberts K., Jaffar Z. (2017). CB2 receptors regulate natural killer cells that limit allergic airway inflammation in a murine model of asthma. Allergy.

[247] Smith S.L., Kennedy P.R., Stacey K.B., Worboys J.D., Yarwood A., Seo S., Solloa E.H., Mistretta B., Chatterjee S.S., Gunaratne P. (2020). Diversity of peripheral blood human NK cells identified by single-cell RNA sequencing. Blood Adv..

[248] Villar J., Segura E. (2020). Decoding the Heterogeneity of Human Dendritic Cell Subsets. Trends Immunol..

[249] Wculek S.K., Cueto F.J., Mujal A.M., Melero I., Krummel M.F., Sancho D. (2020). Dendritic cells in cancer immunology and immunotherapy. Nat. Rev. Immunol..

[250] Do Y., McKallip R.J., Nagarkatti M., Nagarkatti P.S. (2004). Activation through Cannabinoid Receptors 1 and 2 on Den-dritic Cells Triggers Nf-Kappab-Dependent Apoptosis: Novel Role for Endogenous and Exogenous Cannabinoids in Im-munoregulation. J. Immunol..

[251] Matias I., Pochard P., Orlando P., Salzet M., Pestel J., Di Marzo V. (2002). Presence and regulation of the endocannabinoid system in human dendritic cells. JBIC J. Biol. Inorg. Chem..

[252] Maestroni G.J.M. (2004). The endogenous cannabinoid 2-arachidonoyl glycerol as in vivo chemoattractant for dendritic cells and adjuvant for Th1 response to a soluble protein. FASEB J..

[253] Chiurchiù V., Cencioni M.T., Bisicchia E., De Bardi M., Gasperini C., Borsellino G., Centonze D., Battistini L., Maccarrone M. (2013). Distinct modulation of human myeloid and plasmacytoid dendritic cells by anandamide in multiple sclerosis. Ann. Neurol..

[254] Dieu-Nosjean M.-C., Antoine M., Danel C., Heudes D., Wislez M., Poulot V., Rabbe N., Laurans L., Tartour E., De Chaisemartin L. (2008). Long-Term Survival for Patients With Non–Small-Cell Lung Cancer With Intratumoral Lymphoid Structures. J. Clin. Oncol..

[255] Goc J., Germain C., Vo-Bourgais T.K., Lupo A., Klein C., Knockaert S., de Chaisemartin L., Ouakrim H., Becht E., Alifano M. (2014). Dendritic Cells in Tumor-Associated Tertiary Lymphoid Structures Signal a Th1 Cytotoxic Immune Con-texture and License the Positive Prognostic Value of Infiltrating Cd8+ T Cells. Cancer Res..

[256] Ladányi A., Kiss J., Somlai B., Gilde K., Fejős Z., Mohos A., Gaudi I., Tímár J. (2007). Density of DC-LAMP+ mature dendritic cells in combination with activated T lymphocytes infiltrating primary cutaneous melanoma is a strong independent prognostic factor. Cancer Immunol. Immunother..

[257] Garris C.S., Luke J.J. (2020). Dendritic Cells, the T-cell-inflamed Tumor Microenvironment, and Immunotherapy Treatment Response. Clin. Cancer Res..

[258] Sharonov G.V., Serebrovskaya E.O., Yuzhakova D.V., Britanova O.V., Chudakov D.M. (2020). B cells, plasma cells and antibody repertoires in the tumour microenvironment. Nat. Rev. Immunol..

[259] Kemp T.J., Moore J.M., Griffith T.S. (2004). Human B Cells Express Functional Trail/Apo-2 Ligand after Cpg-Containing Oligodeoxynucleotide Stimulation. J. Immunol..

[260] Tao H., Lu L., Xia Y., Dai F., Wang Y., Bao Y., Lundy S.K., Ito F., Pan Q., Zhang X. (2015). Antitumor effector B cells directly kill tumor cells via the Fas/FasL pathway and are regulated by IL-10. Eur. J. Immunol..

[261] Shi J.Y., Gao Q., Wang Z.C., Zhou J., Wang X.Y., Min Z.H., Shi Y.H., Shi G.M., Ding Z.B., Ke A.W. (2013). Margin-Infiltrating Cd20(+) B Cells Display an Atypical Memory Phenotype and Correlate with Fa-vorable Prognosis in Hepatocellular Carcinoma. Clin. Cancer Res..

[262] Erdag G., Schaefer J.T., Smolkin M.E., Deacon D.H., Shea S.M., Dengel L.T., Patterson J.W., Slingluff C.L. (2012). Immunotype and Immunohistologic Characteristics of Tumor-Infiltrating Immune Cells Are Associated with Clinical Outcome in Metastatic Melanoma. Cancer Res..

[263] Wolfson M., Muzzio D., Ehrhardt J., Franchi A., Zygmunt M., Jensen F. (2016). Expression analysis of cannabinoid receptors 1 and 2 in B cells during pregnancy and their role on cytokine production. J. Reprod. Immunol..

[264] Castaneda J.T., Harui A., Kiertscher S.M., Roth J.D., Roth M.D. (2013). Differential Expression of Intracellular and Ex-tracellular Cb(2) Cannabinoid Receptor Protein by Human Peripheral Blood Leukocytes. J. Neuroimmun. Pharmacol..

[265] Basu S., Ray A., Dittel B.N. (2011). Cannabinoid Receptor 2 Is Critical for the Homing and Retention of Marginal Zone B Lineage Cells and for Efficient T-Independent Immune Responses. J. Immunol..

[266] Flygare J., Gustafsson K., Kimby E., Christensson B., Sander B. (2005). Cannabinoid receptor ligands mediate growth inhibition and cell death in mantle cell lymphoma. FEBS Lett..

[267] Wasik A.M., Christensson B., Sander B. (2011). The role of cannabinoid receptors and the endocannabinoid system in mantle cell lymphoma and other non-Hodgkin lymphomas. Semin. Cancer Biol..

[268] Wasik A.M., Almestrand S., Wang X., Hultenby K., Dackland Å.-L., Andersson P., Kimby E., Christensson B., Sander B. (2011). WIN55,212-2 induces cytoplasmic vacuolation in apoptosis-resistant MCL cells. Cell Death Dis..

[269] Gustafsson K., Christensson B., Sander B., Flygare J. (2006). Cannabinoid Receptor-Mediated Apoptosis Induced byR(+)-Methanandamide and Win55,212-2 Is Associated with Ceramide Accumulation and p38 Activation in Mantle Cell Lymphoma. Mol. Pharmacol..

[270] Freund P., Porpaczy E.A., Le T., Gruber M., Pausz C., Staber P., Jäger U., Vanura K. (2016). Cannabinoid Receptors Are Overexpressed in CLL but of Limited Potential for Therapeutic Exploitation. PLoS ONE.

[271] Rosenberg H.F., Dyer K.D., Foster P.S. (2012). Eosinophils: Changing perspectives in health and disease. Nat. Rev. Immunol..

[272] Bagnasco D., Ferrando M., Caminati M., Bragantini A., Puggioni F., Varricchi G., Passalacqua G., Canonica G.W. (2017). Targeting Interleukin-5 or Interleukin-5ralpha: Safety Considerations. Drug Saf..

[273] Mattei F., Andreone S., Marone G., Gambardella A.R., Loffredo S., Varricchi G., Schiavoni G. (2020). Eosinophils in the Tumor Microenvironment. Adv. Exp. Med. Biol..

[274] Klein T.W., Newton C., Larsen K., Lu L., Perkins I., Nong L., Friedman H. (2003). The cannabinoid system and immune modulation. J. Leukoc. Biol..

[275] Oka S., Yanagimoto S., Ikeda S., Gokoh M., Kishimoto S., Waku K., Ishima Y., Sugiura T. (2005). Evidence for the In-volvement of the Cannabinoid Cb2 Receptor and Its Endogenous Ligand 2-Arachidonoylglycerol in 12-O-Tetradecanoylphorbol-13-Acetate-Induced Acute Inflammation in Mouse Ear. J. Biol. Chem..

[276] Oka S., Ikeda S., Kishimoto S., Gokoh M., Yanagimoto S., Waku K., Sugiura T. (2004). 2-Arachidonoylglycerol, an endogenous cannabinoid receptor ligand, induces the migration of EoL-1 human eosinophilic leukemia cells and human peripheral blood eosinophils. J. Leukoc. Biol..

[277] Frei R.B., Luschnig P., Parzmair G.P., Peinhaupt M., Schranz S., Fauland A., Wheelock C.E., Heinemann A., Sturm E.M. (2016). Cannabinoid Receptor 2 Augments Eosinophil Responsiveness and Aggravates Allergen-Induced Pulmonary In-flammation in Mice. Allergy.

[278] Grisaru-Tal S., Itan M., Klion A.D., Munitz A. (2020). A new dawn for eosinophils in the tumour microenvironment. Nat. Rev. Cancer.

[279] Varricchi G., Galdiero M.R., Loffredo S., Lucarini V., Marone G., Mattei F., Marone G., Schiavoni G. (2018). Eosinophils: The unsung heroes in cancer?. OncoImmunology.

[280] Varricchi G., Rossi F.W., Galdiero M.R., Granata F., Criscuolo G., Spadaro G., De Paulis A., Marone G. (2019). Physiological Roles of Mast Cells: Collegium Internationale Allergologicum Update 2019. Int. Arch. Allergy Immunol..

[281] Marone G., Borriello F., Varricchi G., Genovese A., Granata F. (2014). Basophils: Historical Reflections and Perspectives. Chem. Immunol. Allergy.

[282] Schroeder J.T., Chichester K.L., Bieneman A.P. (2009). Human Basophils Secrete IL-3: Evidence of Autocrine Priming for Phenotypic and Functional Responses in Allergic Disease. J. Immunol..

[283] Patella V., Giuliano A., Bouvet J.P., Marone G. (1998). Endogenous superallergen protein Fv induces IL-4 secretion from human Fc epsilon RI+ cells through interaction with the VH3 region of IgE. J. Immunol..

[284] Genovese A., Borgia G., Bjorck L., Petraroli A., de Paulis A., Piazza M., Marone G. (2003). Immunoglobulin Superantigen Protein L Induces Il-4 and Il-13 Secretion from Human Fc Epsilon Ri+ Cells through Interaction with the Kappa Light Chains of Ige. J. Immunol..

[285] De Paulis A., Prevete N., Fiorentino I., Rossi F.W., Staibano S., Montuori N., Ragno P., Longobardi A., Liccardo B., Genovese A. (2006). Expression and Functions of the Vascular Endothelial Growth Factors and Their Receptors in Human Basophils. J. Immunol..

[286] Marone G., Schroeder J.T., Mattei F., Loffredo S., Gambardella A.R., Poto R., De Paulis A., Schiavoni G., Varricchi G. (2020). Is There a Role for Basophils in Cancer?. Front. Immunol..

[287] De Monte L., Reni M., Tassi E., Clavenna D., Papa I., Recalde H., Braga M., Di Carlo V., Doglioni C., Protti M.P. (2011). Intratumor T helper type 2 cell infiltrate correlates with cancer-associated fibroblast thymic stromal lymphopoietin production and reduced survival in pancreatic cancer. J. Exp. Med..

[288] Cencioni M.T., Chiurchiù V., Catanzaro G., Borsellino G., Bernardi G., Battistini L., Maccarrone M. (2010). Anandamide Suppresses Proliferation and Cytokine Release from Primary Human T-Lymphocytes Mainly via CB2 Receptors. PLoS ONE.

[289] Joseph J., Niggemann B., Zaenker K.S., Entschladen F. (2004). Anandamide is an endogenous inhibitor for the migration of tumor cells and T lymphocytes. Cancer Immunol. Immunother..

[290] Gallily R., Breuer A., Mechoulam R. (2000). 2-Arachidonylglycerol, an Endogenous Cannabinoid, Inhibits Tumor Necrosis Factor-Alpha Production in Murine Macrophages, and in Mice. Eur. J. Pharmacol..

[291] Han K.H., Lim S., Ryu J., Lee C.-W., Kim Y., Kang J.-H., Kang S.-S., Ahn Y.K., Park C.-S., Kim J.J. (2009). CB1 and CB2 cannabinoid receptors differentially regulate the production of reactive oxygen species by macrophages. Cardiovasc. Res..

[292] Chouinard F., Lefebvre J.S., Navarro P., Bouchard L., Ferland C., Lalancette-Hébert M., Marsolais D., LaViolette M., Flamand N. (2011). The Endocannabinoid 2-Arachidonoyl-Glycerol Activates Human Neutrophils: Critical Role of Its Hydrolysis and De Novo Leukotriene B4 Biosynthesis. J. Immunol..

[293] Rayman N., Lam K.H., Laman J.D., Simons P.J., Löwenberg B., Sonneveld P., Delwel R. (2004). Distinct Expression Profiles of the Peripheral Cannabinoid Receptor in Lymphoid Tissues Depending on Receptor Activation Status. J. Immunol..

[294] Jorda M.A., SVerbakel E., Valk P.J., Vankan-Berkhoudt Y.V., Maccarrone M., Finazzi-Agro A., Lowenberg B., Delwel R. (2002). Hematopoietic Cells Expressing the Peripheral Cannabinoid Receptor Migrate in Response to the Endocanna-binoid 2-Arachidonoylglycerol. Blood.

[295] Tanikawa T., Kurohane K., Imai Y. (2007). Induction of Preferential Chemotaxis of Unstimulated B-Lymphocytes by 2-Arachidonoylglycerol in Immunized Mice. Microbiol. Immunol..

[296] Guzmán M. (2003). Cannabinoids: Potential anticancer agents. Nat. Rev. Cancer.

[297] Laezza C., Pagano C., Navarra G., Pastorino O., Proto M.C., Fiore D., Piscopo C., Gazzerro P., Bifulco M. (2020). The Endocannabinoid System: A Target for Cancer Treatment. Int. J. Mol. Sci..

[298] Kienzl M., Kargl J., Schicho R. (2020). The Immune Endocannabinoid System of the Tumor Microenvironment. Int. J. Mol. Sci..

[299] Ciaglia E., Torelli G., Pisanti S., Picardi P., D’Alessandro A., Laezza C., Malfitano A.M., Fiore D., Zottola A.C.P., Proto M.C. (2015). Cannabinoid receptor CB1 regulates STAT3 activity and its expression dictates the responsiveness to SR141716 treatment in human glioma patients’ cells. Oncotarget.

[300] Utomo W.K., De Vries M., Braat H., Bruno M.J., Parikh K., Comalada M., Peppelenbosch M., Van Goor H., Fuhler G.M. (2017). Modulation of Human Peripheral Blood Mononuclear Cell Signaling by Medicinal Cannabinoids. Front. Mol. Neurosci..

[301] Lavin Y., Kobayashi S., Leader A., Amir E.-A.D., Elefant N., Bigenwald C., Remark R., Sweeney R., Becker C.D., Levine J.H. (2017). Innate Immune Landscape in Early Lung Adenocarcinoma by Paired Single-Cell Analyses. Cell.

[302] Braga F.A.V., Kar G., Berg M., Carpaij O.A., Polanski K., Simon L.M., Brouwer S., Gomes T., Hesse L., Jiang J. (2019). A cellular census of human lungs identifies novel cell states in health and in asthma. Nat. Med..

[303] Hua T., Li X., Wu L., Iliopoulos-Tsoutsouvas C., Wang Y., Wu M., Shen L., Brust C.A., Nikas S.P., Song F. (2020). Activation and Signaling Mechanism Revealed by Cannabinoid Receptor-Gi Complex Structures. Cell.

[304] Xing C., Zhuang Y., Xu T.-H., Feng Z., Zhou X.E., Chen M., Wang L., Meng X., Xue Y., Wang J. (2020). Cryo-EM Structure of the Human Cannabinoid Receptor CB2-Gi Signaling Complex. Cell.

